# Arginine 65 methylation of Neurogenin 3 by PRMT1 is required for pancreatic endocrine development of hESCs

**DOI:** 10.1038/s12276-023-01035-8

**Published:** 2023-07-03

**Authors:** Gahyang Cho, Kwangbeom Hyun, Jieun Choi, Eunji Shin, Bumsoo Kim, Hail Kim, Jaehoon Kim, Yong-Mahn Han

**Affiliations:** 1 Department of Biological Sciences, Korea Advanced Institute of Science and Technology, Daejeon, 34141 Republic of Korea; 2 Graduate School of Medical Science and Engineering, Korea Advanced Institute of Science and Technology, Daejeon, 34141 Republic of Korea

**Keywords:** Embryonic stem cells, Methylation

## Abstract

Neurogenin 3 (NGN3) is a key transcription factor in the cell fate determination of endocrine progenitors (EPs) in the developing pancreas. Previous studies have shown that the stability and activity of NGN3 are regulated by phosphorylation. However, the role of NGN3 methylation is poorly understood. Here, we report that protein arginine methyltransferase-1 (PRMT1)-mediated arginine 65 methylation of NGN3 is required for the pancreatic endocrine development of human embryonic stem cells (hESCs) in vitro. We found that inducible PRMT1-knockout (P-iKO) hESCs did not differentiate from EPs into endocrine cells (ECs) in the presence of doxycycline. Loss of PRMT1 caused NGN3 accumulation in the cytoplasm of EPs and decreased the transcriptional activity of NGN3. We found that PRMT1 specifically methylates NGN3 arginine 65 and that this modification is a prerequisite for ubiquitin-mediated degradation. Our findings demonstrate that arginine 65 methylation of NGN3 is a key molecular switch in hESCs permitting their differentiation into pancreatic ECs.

## Pancreas: Development of hormone-producing cells

Examination of the endocrine (hormone-producing) cells of the pancreas reveals how the modification of a key regulatory protein acts as a molecular switch to ensure the development of endocrine cells. In the developing pancreas, the transcription factor neurogenin 3 (NGN3) plays a critical role in triggering differentiation of human embryonic stem cells (hESCs) into endocrine cells. NGN3 then degrades rapidly, but the exact details of this process are unclear. Using human cell lines, Yong-Mahn Han and co-workers at the Korea Advanced Institute of Science and Technology, Daejeon, South Korea, demonstrated that a particular amino acid on NGN3 is the target for the protein arginine methyltransferase-1 (PRMT1). PRMT1 methylates arginine 65, and this modification permits the expression of certain genes before triggering NGN3 degradation. Stem cells without PRMT1 failed to differentiate, and NGN3 accumulated in the cell cytoplasm rather than degrading.

## Introduction

The endocrine system of the pancreas—a small proportion (1-2%) of the pancreas as a whole—comprises small globular clusters of specialized cells, such as the islets of Langerhans^1^. In mammalian embryos, the cells of the pancreatic endocrine lineage develop through a series of successive stages, including the definitive endoderm (DE), pancreatic endoderm (PE), endocrine progenitor (EP), and endocrine cell (EC) stages^2,3^. As PEs arise from DEs, they begin to exhibit strong expression of pancreatic and duodenal homeobox-1 (PDX1) at E8.5 in mice and 30 d post-conception in humans^4^. Small populations of PDX1-positive endoderm cells then differentiate into highly heterogeneous EPs^5^ and exhibit transient activation of the transcription factor Neurogenin 3 (NGN3)^6–8^. NGN3 is a critical determinant of EC fate that activates EC-associated genes^9,10^. NGN3^NULL^ mice fail to develop any pancreatic EC lineages^11^. Normally, the high NGN3 expression of trunk EPs falls abruptly within a few days^12^. Although it is clear that multiphosphorylation of NGN3 facilitates both its ubiquitin-mediated degradation and its transcriptional activity^13,14^, it is unknown whether other more elusive modifications important for NGN3 degradation or stability are involved in pancreatic development.

Protein arginine methyltransferase-1 (PRMT1), the predominant methyltransferase in mammalian cells, methylates arginine residues in conserved glycine and arginine-rich regions of histone and nonhistone proteins^15,16^. Through its role in arginine methylation, PRMT1 contributes to diverse cellular processes, such as epithelial–mesenchymal transition, the cell cycle, DNA repair, and RNA processing^17–20^. It also functions as a transcriptional coactivator by generating asymmetrical dimethyl-arginine on histone H4 (H4R3me2a)^21,22^. PRMT1-mediated arginine methylation plays an important role in the stability and localization of nonhistone proteins, such as FOXO1, TSC2, and progesterone receptor^23–25^. Recently, Prmt1-knockout mouse embryos showed slowed degradation of NGN3 from E14.5, leading to severe postnatal pancreatic hypoplasia^26^. This suggests that PRMT1 is associated with NGN3 stability in pancreatic development. Nonetheless, the mechanism by which PRMT1 affects pancreatic development via its regulation of the stability and activity of NGN3 is poorly understood. The aim of this study was to investigate the molecular mechanism by which PRMT1 affects NGN3 and its role in human pancreatic EC development.

## Materials and methods

### Plasmid constructs for the P-iKO system and NGN3 expression

The ZFN-L, ZFN-R, pAAV-Neo_Cas9, and pAAV-Puro_siKO vectors^27,28^ were provided by Prof. KJ Yoon at the Department of Biological Sciences, KAIST. The pAAV-Puro_siKO vector was introduced with a chimeric sgRNA expression cassette using the AarI enzyme (New England Biolabs, Ipswich, MA). The sgRNA 5′-ATGTGACGGCCATCGAGGAC-3′ (PAM: CGG) was recommended by IDT CRISPR_PREDESIGN (Integrated DNA Technologies, Inc., IA). For expression of human NGN3, either full-length or fragments of NGN3 cDNA were inserted into the pCAG-FLAG-IPuro or pGEX4T-1 vectors, respectively.

### Generation of the P-iKO hESC line

H1 hESCs were transfected with ZFN-L (5 μg), ZFN-R (5 μg), pAAV-Neo_Cas9 (2.5 μg), and pAAV-Puro_siKO (2.5 μg) plasmids with a NEPA21 Super Electroporator (Nepa Gene, Chiba, Japan; poring pulse: 150 V, transfer pulse: 20 V). The transfected cells were placed on a Matrigel-coated culture dish and incubated in mTeSR1 medium (STEMCELL Technologies Inc., Vancouver, Canada) containing 10 μM Y27632 (ROCK inhibitor, A. G. Scientific, San Diego, CA) at 37 °C and 5% CO_2_ for 3 d until they reached confluence. For transgenic colony selection, the cells were treated with 200 μg/ml G418 (InvivoGen, Pak Shek Kok, Hong Kong) for 4 d and then with 0.5 μg/ml puromycin (Sigma, St. Louis, MO) for 3 d. To expand the hESCs, each ESC colony was mechanically divided into 20–25 clumps, treated with 10 μg/ml dispase (Invitrogen, Carlsbad, CA) at 37 °C for 4 min, and divided into 3–4 culture dishes. All hESCs used in this study were mycoplasma-negative.

### Differentiation of hESCs into pancreatic endocrine cells

hESC colonies were dissociated into single cells with 0.5 μM EDTA at 37 °C for 6 min. Then, 6.5 × 10^4^ cells were incubated on Matrigel-coated 4-well plates (SPL Lifesciences, Pocheon, Korea) in 500 μl of mTeSR1 medium supplemented with 10 μM Y27632 at 37 °C and 5% CO_2_ for 2 d. hESCs were differentiated into pancreatic ECs as previously reported^29^.

### Electroporation

Before electroporation, P-iKO PEs were preincubated in PE medium containing 10 μM Y27632 for 1 h. The cells were rinsed with DPBS and detached by Accutase (Innovative Cell Technologies, Inc., CA) at 37 °C for 10 min. The dissociated cells were centrifuged at room temperature (RT) at 300 × *g* for 3 min. After two washes in 1× Opti-MEM (Invitrogen), 1 × 10^6^ cells and 10 μg of DNA vector were mixed and electroporated at 150 V for 2.5 msec while suspended in 100 μl of Opti-MEM in a 2 mm-gap cuvette. Electroporated cells were cultured in PE medium supplemented with 10 μM Y27632 at 37 °C and 5% CO_2_ for 1–2 d.

### Western blot analysis

Cultured cells were collected using a scraper and lysed in RIPA buffer (InvivoGen) containing protease and phosphatase inhibitors. The lysates were sonicated briefly and cellular debris was removed by centrifugation at 16,000 × *g* at 4 °C for 25 min. The total protein concentration was calculated via Bradford assay (Bio-Rad, Hercules, CA). Ten micrograms of total protein was separated by SDS‒PAGE electrophoresis and transferred to a nitrocellulose membrane with either 0.4 μm (GE Healthcare, Chicago, IL) or 0.2 μm pores (Invitrogen). After blocking with 5% BSA or 4% skim milk in TBST (10 mM Tris-HCl [pH 7.5], 150 nM NaCl, and 0.1% Tween 20), the membranes were incubated in 5% BSA or 4% skim milk TBST at 4 °C overnight with the appropriate primary antibodies (Table 2). After three rinses with TBST, the membranes were incubated in 4% skim milk TBST at RT for 1 h with anti-mouse or anti-rabbit IgG HRP-linked secondary antibodies (CST). The protein bands were visualized in ECL solution (Merck KGaA, Darmstadt, Germany) using an ImageQuant LAS4000 (GE Healthcare). The band intensities were quantified using ImageJ (NIH).

### Quantitative RT‒PCR

Total mRNA was extracted from cultured cells using Easy-BLUE solution (Intron Biotechnology, Seongnam, Korea) and reverse-transcribed with a cDNA synthesis kit (SolGent Co., Ltd., Daejeon, Korea). Quantitative RT‒PCR was carried out with SYBR Green and Taq DNA polymerase (SolGent) on a Bio-Rad CFX96 Real-Time System (Bio-Rad). For analysis of relative expression, the Cq value for each gene was normalized to the GAPDH mRNA level using the ΔΔCq method. The primer sequences are listed in Table 1.

**Table 1:** Quantitative RT‒PCR primer list.

Target	5′-Forward	5′-Reverse	bp
GAPDH	CTTCGCTCTCTGCTCCTCCT	GTTAAAAGCAGCCCTGGTGA	154
PRMT1	ATCGAGGACCGGCAGTACAA	CTAGGGGCTCCTTAATGGCC	103
OCT4	TCGGGGTGGAGAGCAACT	GGGTGATCCTCTTCTGCTTC	177
SOX2	ACCAGCTCGCAGACCTACAT	TGGAGTGGGAGGAAGAGGTA	154
FOXA2	AACAAGATGCTGACGCTGAG	CAGGAAACAGTCGTTGAAGG	126
CXCR4	GGTGGTCTATGTTGGCGTCT	TGGAGTGTGACAGCTTGGAG	227
SOX17	CAGAATCCAGACCTGCACAA	GCGGCCGGTACTTGTAGTT	154
GATA4	TCCAAACCAGAAAACGGAAG	CTGTGCCCGTAGTGAGATGA	187
PDX1	GTTCCGAGGTAGAGGCTGTG	AACATAACCCGAGCACAAGG	250
SOX9	AGCGAACGCACATCAAGAC	GCTGTAGTGTGGGAGGTTGAA	110
HNF1b	AGCCCACCAACAAGAAGATG	CATTCTGCCCTGTTGCATTC	145
NGN3	GGCTGTGGGTGCTAAGGGTAAG	CAGGGAGAAGCAGAAGGAACAA	104
NEUROD1	GTTCTCAGGACGAGGAGCAC	GTCTCTTGGGCTTTTGATCG	168
PAX6	GTGTCCAACGGATGTGTGAG	CTAGCCAGGTTGCGAAGAAC	254
PAX4	CTGGCTACACCCCCTGTG	TCCCTGGTCCTCCTGTAATG	174
NKX2.2	TGGCCATGTAAACGTTCTGA	GGAAGAAAGCAGGGGAAAAC	189
INS	TGTACCAGCATCTGCTCCCTCTA	TGCTGGTTCAAGGGCTTTATTCCA	122
GCG	AGGCAGACCCACTCAGTGA	AACAATGGCGACCTCTTCTG	308
SST	CCCCAG ACT CCG TCA GTTTC	TCC GTC TGG TTG GGT TCAG	108
PPY	ACCTGCGTGGCTCTGTTACT	TACCTAGGCCTGGTCAGCAT	152
CHGA	CCTGTCAGCCAGGAATGTTT	CATCCTTGGATGATGGCTCT	235

### Immunostaining

Cells were fixed with 4% formaldehyde (Sigma) at RT for 30 min. After three rinses in PBS, the cells were incubated in PBS containing 0.1% Triton-X (Sigma) for 30 min and blocked in 1% BSA (Sigma) for 1 h. After the addition of the appropriate primary antibodies (Table 2), the cells were incubated in blocking solution at 4 °C overnight. After three washes, the cells were treated with Alexa Fluor 488- or 594-conjugated donkey antibodies (Abcam) at a dilution ratio of 1:1000 and further incubated in blocking solution at RT for 1 h. DAPI (Invitrogen) was used for nuclear counterstaining. After five washes in TBST, the stained cells were observed.

**Table 2:** Primary antibodies.

Target	Manufacturer	Cat. #	Host	Dilution factor
PRMT1	Abcam	ab70724	Rb	WB 1:1000, IF 1:1000
NGN3	DSHB	F25A1B3	m	WB 1:500, IF 1:200
FLAG	Santa Cruz	sc-51590	m	WB 1:500
ADMA	Cell Signaling Technology	13522S	Rb	WB 1:500
monoR	ImmuneChem Pharmaceuticals	ICP0801	Rb	WB 1:500
HRP-conjugated GAPDH	Santa Cruz	sc-47724	m	WB 1:2000
H4R3me2a	Abcam	ab194683	Rb	WB 1:1000, IF 1:1000
Histone H3	Cell Signaling Technology	9715s	Rb	WB 1:2000
SOX2	Cell Signaling Technology	3579S	Rb	IF 1:200
TRA 1–60	Millipore	MAB4360	m	IF 1:200
TRA 1–81	Millipore	MAB4381	m	IF 1:200
OCT4	Santa Cruz	sc-8628	goat	IF 1:500
SOX17	R&D Systems	AF1924	goat	IF 1:400
GATA4	Santa Cruz	sc-25310	m	IF 1:400
PDX1	Abcam	ab47267	Rb	IF 1:1000
HNF1b	Santa Cruz	sc-7411	goat	IF 1:200
HNF4a	Santa Cruz	sc-8987	Rb	IF 1:400
SOX9	Abcam	ab185966	Rb	IF 1:2000
insulin	DAKO	A0564	Guinea pig	IF 1:1000
c-peptide	Abcam	ab8297	m	IF 1:1000
pancreatic polypeptide	R&D Systems	MAB62971	m	IF 1:200
somatostatin	DAKO	A0566	Rb	IF 1:1000
NEUROD1	Abcam	ab16508	Rb	WB 1:1000
NKX2.2	DSHB	74.5A5	m	IF 1:100

### Immunoprecipitation

Transfected cells were sonicated in cold BC300 buffer (20 mM Tris [pH 7.9], 0.2 mM EDTA, 20% glycerol, 300 mM KCl, 0.1% Tween 20, and 0.2 mM PMSF). After centrifugation at 16,000 × *g* at 4 °C for 25 min, the supernatants were aliquoted, 5% (v/v) for an input sample and 95% (v/v) for immunoprecipitation. The 95% (v/v) lysate aliquots were incubated with a monoclonal anti-FLAG M2 antibody (Sigma) at 4 °C overnight with mild agitation. Then, FLAG-M2 conjugates were washed three times in 700 μl of cold BC300 buffer. FLAG-NGN3 was eluted using FLAG peptide. For immunoprecipitation of FLAG-NGN3 with HA-Ub, transfected HEK cells were treated with a proteasome inhibitor (20 μM MG132, Selleck Chemicals, TX) for 2 h. The eluted samples were boiled at 95 °C for 5 min and analyzed by western blotting.

### siRNA transfection

HEK cells were transfected with the appropriate siRNAs using Lipofectamine RNAiMAX reagent (Invitrogen) according to the manufacturer’s instructions. This study used a human PRMT1-specific siRNA (cat#sc-41069, Santa Cruz) and a nontargeting control siRNA (Dharmacon, Lafayette, CO).

### Purification of recombinant proteins

To produce GST-tagged proteins, cDNAs encoding human NGN3 protein fragments were subcloned into pGEX4T-1 (GE Healthcare), expressed in *Escherichia coli*, and purified on Glutathione-Sepharose 4B beads (GE Healthcare) as previously described^30^. For FLAG-tagged proteins, cDNAs encoding mouse PRMT proteins were subcloned into pFASTBAC1 (Invitrogen) with a FLAG tag. Baculoviruses with individual mouse PRMT cDNAs were generated according to the manufacturer’s instructions (Invitrogen). These were then used to infect Sf9 insect cells. The infected Sf9 cells were incubated in Grace’s insect medium (Invitrogen) supplemented with 10% FBS at 27 °C for 3 d. Finally, proteins were purified from the infected cells on M2 agarose (Sigma) as previously described^30^.

### In vitro methyltransferase assays

Purified substrates (i.e., 400 ng histone octamer or NGN3 proteins and 200 or 400 ng PRMT proteins) were mixed and then incubated in 20 μl of reaction buffer (25 mM HEPES at pH 7.6, 50 mM KCl, 5 mM MgCl_2_, and 4 mM DTT) supplemented with 200 μM cold SAM (*S*-adenosyl methionine) (NEB; for immunoblotting) or 1 µCi ^3^H-labeled SAM (PerkinElmer; for fluorography) at 37 °C for 60 min. The proteins were resolved by 15% SDS‒PAGE and subjected to immunoblotting (cold SAM) or autoradiography (radiolabeled SAM). Anti-dimethyl (asymmetrical) arginine (ICP0810) and anti-monomethyl arginine (ICP0801) antibodies were purchased from ImmuneChem.

### Luciferase reporter assays

After 1d incubation in DMEM containing 10% FBS, HEK cells were transfected with FLAG-NGN3 WT or R65A mutant-expressing vectors, the pGL3-NEUROD1 promoter (~1 kb) firefly luciferase reporter, and a Renilla luciferase-expressing vector at a 1:10:1 ratio using Lipofectamine 2000 (Invitrogen). The transfected cells were cultured in the same medium for 2 d. Luciferase assays were performed using a Dual-Luciferase® Reporter Assay System kit (Promega Corporation, Madison, WI). The firefly luciferase levels were normalized to the Renilla luciferase levels.

### NGN3 degradation assays

HEK cells were transfected with the FLAG-NGN3 vector and then incubated in DMEM containing 10% FBS for 1d. After changing to fresh medium supplemented with 10 μg/ml cycloheximide (CHX, Sigma), the transfected cells were immediately lysed with cold RIPA buffer containing protease and phosphatase inhibitors at each time point.

### Statistical analysis

Statistical analyses of all data were carried out using GraphPad Prism 7 (GraphPad Software, Inc., La Jolla, CA). Each experiment was performed at least in triplicate. The data are presented as the means ± SEMs. Statistical significance was calculated via Student’s *t* tests.

### Ethics statement

The Korea Advanced Institute of Science and Technology IRB approved the usage of hESCs described in this manuscript (KH2021-191).

## Results

### Generation of a P-iKO hESC line

**Fig. 1:**
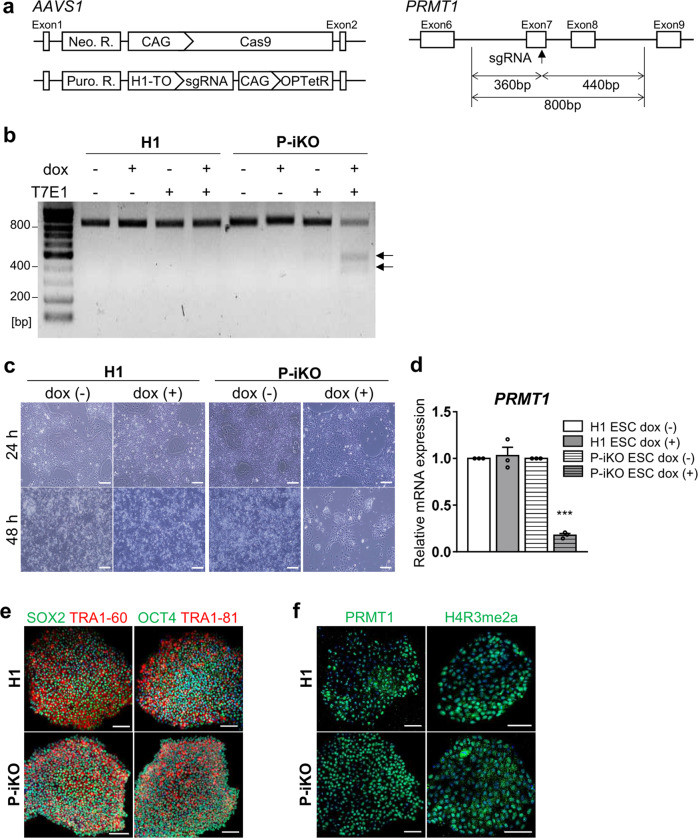
Generation of a P-iKO hESC line. **a** *AAVS1* locus-targeted alleles for the generation of the P-iKO hESC line (left). Schematic of the *PRMT1* exon targeted using the doxycycline-inducible CRISPR system (right). Neo. R., Neomycin resistance gene; Puro. R., puromycin resistance gene; H1-TO, tetracycline-inducible H1 RNA Polymerase III promoter containing a tetO2 sequence; OPTetR, OPTimized Tetracycline-responsive Repressor^28^. See also Supplementary Fig. 1. **b** T7E1 analysis of genomic DNA from P-iKO hESCs. A single transgenic cell line after dox treatment was identified by the two genomic DNA fragments indicated by arrows (at 360 and 440 bp). **c** P-iKO hESC morphology. Most P-iKO hESCs died within 48 h of dox treatment. Scale bars, 200 μm. **d** Relative *PRMT1* mRNA levels in P-iKO hESCs. The relative expression values are presented as the mean ± SEM (*n* = 3). ***p < 0.001. **e** Normal expression of pluripotency-associated markers in P-iKO hESCs. Scale bars, 100 μm. **f** Immunostaining of PRMT1 and H4R3me2a in P-iKO hESCs. Scale bars, 100 μm.

To understand the role of arginine methylation in pancreatic endocrine cell development, we generated a PRMT1-inducible knockout (P-iKO) hESC line (Supplementary Fig. 1). One *AAVS1* allele was targeted for constitutive Cas9 expression, and the other was targeted for tetracycline-dependent (TetR) sgRNA transcription (Fig. 1a, left panel). We designed the sgRNA to target human *PRMT1* exon 7, which encodes the middle part of the SAM-dependent methyltransferase domain (Fig. 1a, right panel). In normal media, P-iKO cells maintain an intact *PRMT1* genomic locus because they produce only the Cas9 protein. The P-iKO cells begin expressing the sgRNA when exposed to doxycycline (dox). Then, the Cas9-sgRNA complex makes indels in the *PRMT1* locus of P-iKO cells (Fig. 1b, arrows: 360, 440 bp). In the presence of dox, P-iKO hESCs died within 48 h, whereas H1 hESCs survived (Fig. 1c). As expected, *PRMT1* transcript levels were significantly lower in dox-treated P-iKO hESCs than in untreated ones (Fig. 1d). However, similar to H1 hESCs, P-iKO hESCs without dox treatment exhibited normal expression of pluripotency markers, including SOX2, TRA 1-60, OCT4, and TRA 1–81 (Fig. 1e). They also showed normal expression of PRMT1 and methylation of histone 4 (H4R3me2a) in the absence of dox (Fig. 1f). Thus, we generated pluripotent P-iKO hESCs with an inducible P-KO system in vitro.

### P-iKO hESCs were differentiated into pancreatic endocrine cells in the absence of doxycycline

**Fig. 2:**
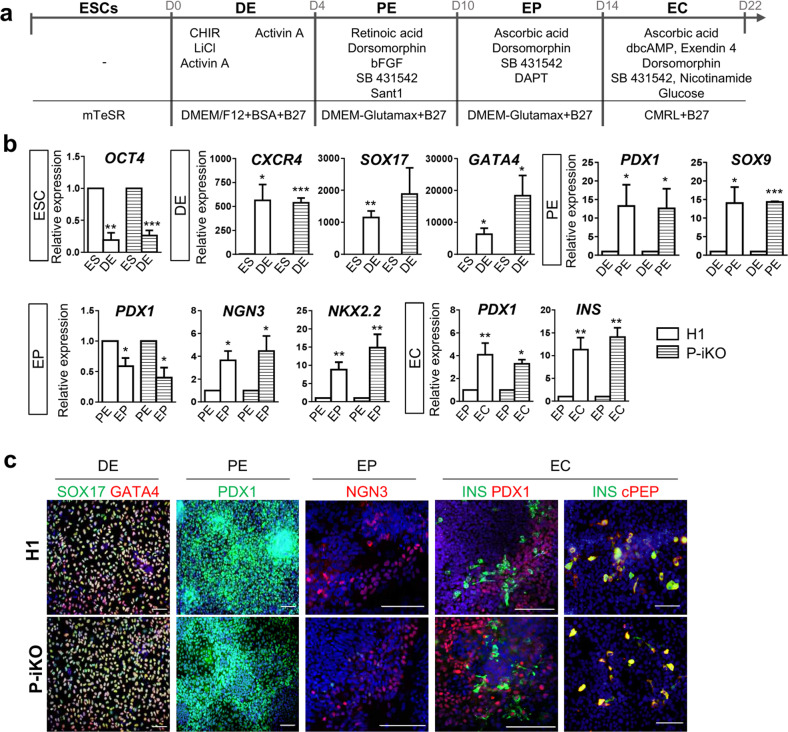

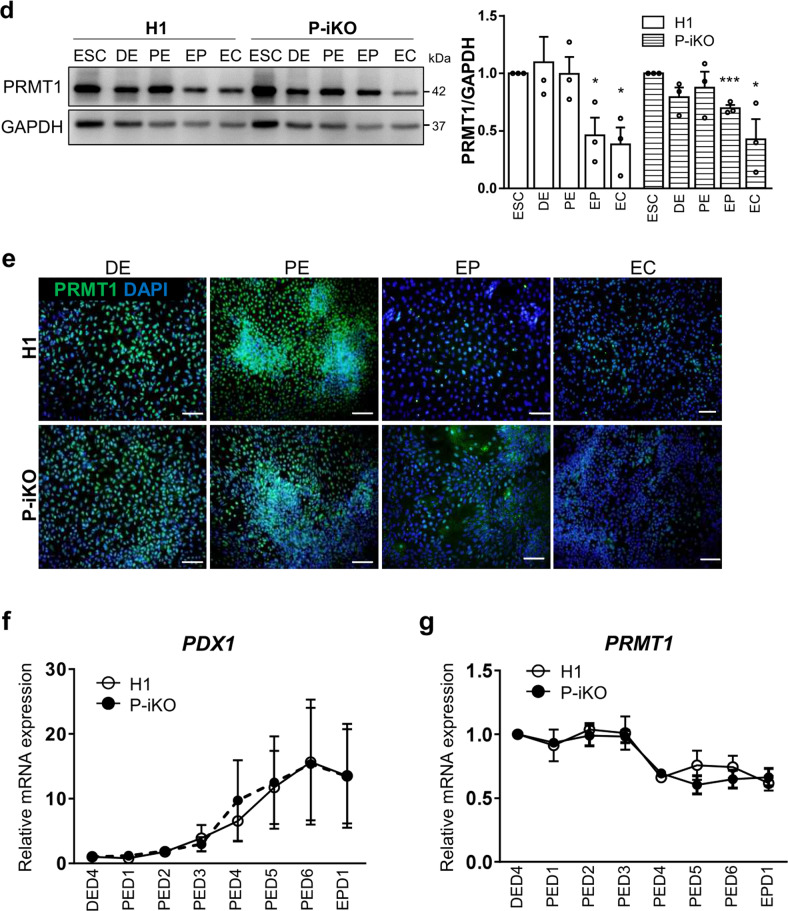
Differentiation of P-iKO hESCs into pancreatic endocrine cells. **a** Overall protocol for the differentiation of hESCs into pancreatic endocrine cells. The detailed procedures are described in the “Materials and methods” section. D, day. See also Supplementary Fig. 2a. **b** Transcript expression of developmental genes in P-iKO hESCs in the absence of dox treatment during pancreatic differentiation. The relative expression values are presented as the mean ± SEM. *p < 0.05, **p < 0.01, ***p < 0.001 (*n* = 3–4). See also Supplementary Fig. 2b. **c** Immunostaining of pancreatic developmental markers in P-iKO hESCs undergoing pancreatic differentiation. Scale bars, 100 μm. **d** Expression of PRMT1 protein in P-iKO hESCs as they progressed through the stages of pancreatic differentiation. The relative expression values are presented as the mean ± SEM. *p < 0.05, ***p < 0.001 (*n* = 3). **e** Immunostaining of PRMT1 in P-iKO hESCs as they progressed through the stages of pancreatic lineage differentiation. Scale bars, 100 μm. **f** Transcriptional profiles of the *PDX1* gene as P-iKO hESCs and controls progressed from Day 4 of the DE stage (DED4) to Day 1 of the EP stage (EPD1) of pancreatic lineage differentiation. The data are presented as the mean ± SEM (*n* = 6). **g** Transcriptional profiles of the *PRMT1* gene in P-iKO hESCs and controls progress from Day 4 of the DE stage (DED4) to Day 1 of the EP stage (EPD1) of pancreatic lineage differentiation. The data are represented as the mean ± SEM (*n* = 5).

The protocol we used to differentiate human ESCs into pancreatic endocrine cells (ECs) is depicted in Fig. 2a. We found that H1 and P-iKO hESCs both differentiated normally into definitive endoderm (DE), pancreatic endoderm (PE), endocrine progenitor (EP), and ECs according to appearance (Supplementary Fig. 2a). In the absence of dox, we did not observe any difference between H1 and P-iKO hESCs in the transcription of genes associated with each developmental stage (Fig. 2b). We also observed similar expression of the same markers in each developmental stage in an immunostaining in H1 and P-iKO hESCs (Fig. 2c and Supplementary Fig. 2b). In the absence of dox, the expression of PRMT1 was similar in each group, falling significantly from the EP stage (Fig. 2d, e). To determine the timing of dox treatment for P-KO induction, we examined *PDX1* expression daily. In both H1 and P-iKO hESCs, *PDX1* transcript levels gradually increased from Day 3 of PE stage (PED3) and then decreased upon reaching the EP stage (Fig. 2f). During the same period, the transcription profiles of *PRMT1* also remained similar in both groups (Fig. 2g). Thus, after confirming the similarity of H1 and P-iKO hESCs in the absence of dox, for all subsequent experiments, we added dox to the PE medium beginning on PED3 for an additional 3 d to knockout PRMT1 (Supplementary Fig. 2c). After dox treatment, P-iKO cells had normal morphologies at the PE and EP stages but showed relatively flattened shapes at the EC stage compared to those of the non-treated cells (Supplementary Fig. 2d). In addition, dox treatment did not influence the cell number or viability in P-iKO PEs, nor did it influence the cell cycle or the number of EPs (Supplementary Fig. 2e, f). Collectively, these results confirm that pluripotent P-iKO hESCs differentiate into pancreatic ECs.

### PRMT1-KO impairs the function of NGN3 in pancreatic endocrine development

**Fig. 3:**
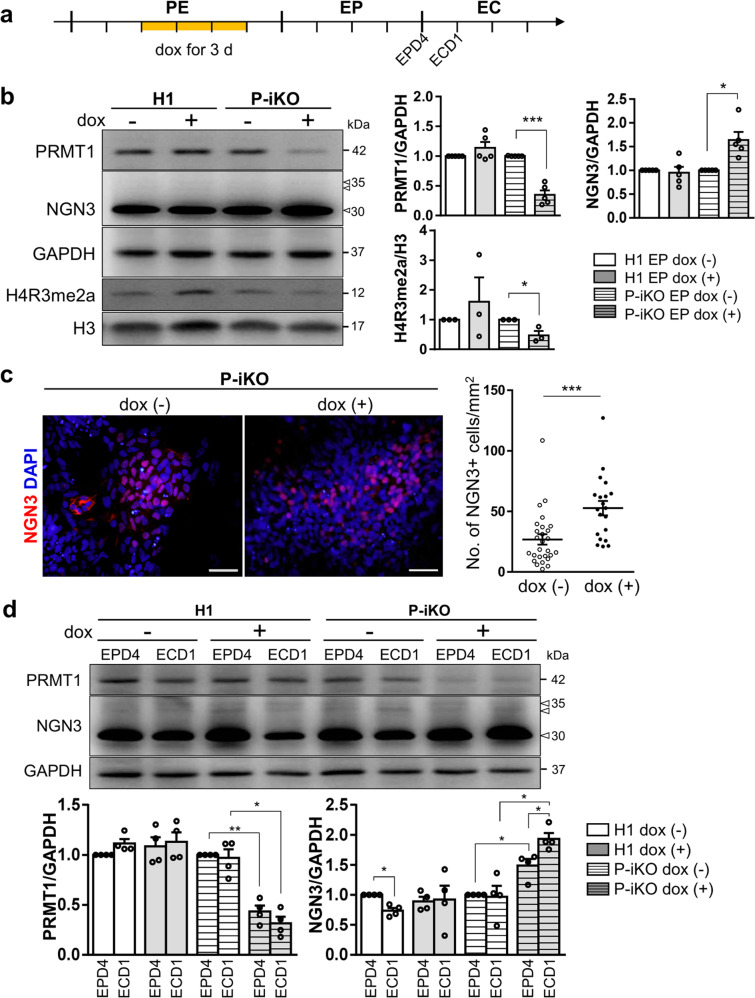

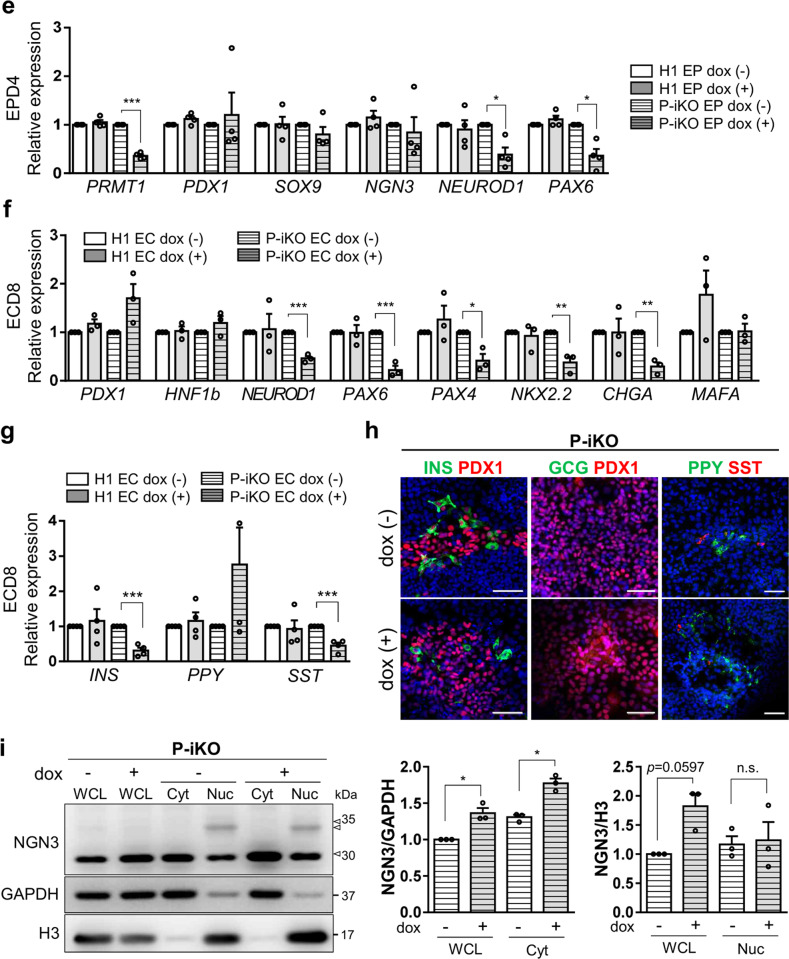
Effects of PRMT1-KO on the differentiation of pancreatic EPs into ECs. **a** Schedule for the dox treatment of PEs differentiated from P-iKO hESCs (yellow line). Dox was administered for 3 d to PRMT1-iKO hESCs that had progressed to D3 of the PE stage of pancreatic lineage development. See also Supplementary Fig. 2d–f. **b** Expression of PRMT1 and NGN3 in P-iKO EPs treated with dox. Relative PRMT1 and NGN3 protein expression levels were normalized to GAPDH levels. The H4R3me2a expression levels were normalized to H3 levels. The data are presented as the mean ± SEM. *p < 0.05, ***p < 0.001 (*n* = 3–5). See also Supplementary Fig. 3a. **c** Immunostaining of NGN3 in P-iKO EPs treated with dox (left). NGN3-positive cells were counted using ImageJ (right). P-KO EPs accumulate NGN3. The data are presented as the mean ± SEM. ***p < 0.001. Dox(−), *n* = 27; dox(+), *n* = 20. Scale bars, 50 μm. See also Supplementary Figs. 3–5. **d** Expression of PRMT1 and NGN3 in P-iKO EPs treated with dox. Western blot analysis showed reduced PRMT1 and enhanced NGN3 levels in P-KO EPD4 and ECD1 cells. PRMT1 and NGN3 protein expression levels were normalized to those of GAPDH. Dox(+) and dox(-) indicate treated and untreated cells, respectively. The data are presented as the mean ± SEM. *p < 0.05, **p < 0.01 (*n* = 4). **e** Relative mRNA expression of EP-associated genes in P-KO EPs. The transcription of NGN3 target genes was significantly downregulated in P-KO EPs. The data are presented as the mean ± SEM. *p < 0.05, ***p < 0.001 (*n* = 4). See also Supplementary Fig. 3d–g. **f** Relative mRNA expression of EC-associated genes in P-KO ECs. Transcripts of NGN3 target genes and EC-associated genes were significantly decreased in P-KO ECs. The data are presented as the mean ± SEM. *p < 0.05, **p < 0.01, ***p < 0.001 (*n* = 3). See also Supplementary Fig. 3h, i. **g** Relative mRNA expression of endocrine hormone genes in P-KO ECs. *INS* and *SST* genes were transcriptionally downregulated in P-KO ECs. The data are presented as the mean ± SEM. ***p < 0.001 (*n* = 4). INS, insulin; PPY, pancreatic polypeptide; SST, somatostatin. **h** Immunostaining of endocrine hormones in P-KO ECs. GCG-positive cells were not observed. Scale bars, 50 μm. **i** Cytoplasmic and nuclear fractionation of endogenous NGN3 in P-KO EPs. P-KO EPs accumulated NGN3 in the cytoplasm. The data are presented as the mean ± SEM. WCL, whole-cell lysate; Cyt, cytoplasm; Nuc, nucleus; n.s., not significant. *p < 0.05 (*n* = 3). See also Supplementary Figs. 4e, 5.

NGN3 is an essential transcription factor for endocrine cell fate decisions and has a very short half-life^31^. We confirmed the EP stage-specific NGN3 expression in both H1 and P-iKO hESCs during in vitro pancreatic endocrine differentiation, observing robust expression only in the EP stage that fell abruptly by the EC stage (Supplementary Fig. 3a). To determine whether P-KO was also associated with NGN3 accumulation in this study, we treated P-iKO cells with dox in the PE for 3 d and further differentiated them into EPs (Fig. 3a). In the presence of dox, we observed reduced expression of PRMT1 (*p* < 0.001) but enhanced expression of NGN3 (*p* < 0.05) in EPs (Fig. 3b). We observed reduced H4R3me2a in P-KO cells compared to untreated cells (Fig. 3b) and more NGN3-positive P-KO cells than untreated P-iKO cells (*p* < 0.001, Fig. 3c). This increase in NGN3-positive P-KO cells continued into the EC stage (Supplementary Fig. 3b, c). Thus, we demonstrated that P-KO increases the proportion of NGN3-positive cells in hESCs undergoing pancreatic development.

In general, NGN3 protein levels increased in the EP stage and then decreased in the EC stage as hESCs progressed through differentiation (Supplementary Fig. 3a). Next, we investigated whether P-KO is associated with NGN3 stability in the transition from EPs to ECs. Normally, NGN3 protein expression was highest in EPD4 and decreased in ECD1. However, P-KO ECD1 showed greater protein expression than EPD4 (Fig. 3d). As a transcription factor, NGN3 activates the expression of pancreatic developmental genes, including *NEUROD1* and *PAX6*^8,32,33^. We were surprised to find that P-iKO cells treated with dox showed lower expression of *NEUROD1* and *PAX6* than untreated cells, although we did not observe any differences in the expression levels of the pancreatic developmental genes *PDX1, SOX9*, and *NGN3* (Fig. 3e). In addition, reduced NEUROD1 expression was confirmed at the protein level in dox(+) P-iKO EPs, but the expression of NKX2.2 and NGN3 nontarget genes, such as PDX1, was not affected (Supplementary Fig. 3d–g). To clarify the reduced NGN3 activity in NGN3-positive EPs, NGN3-T2A-EGFP hESCs were generated from P-iKO hESCs (Supplementary Fig. 4). EGFP-reporter revealed that FACS-sorted EGFP-positive EPs showed significantly lower *NEUROD1* mRNA expression in the dox(+) condition than in the dox(−) condition. Considering the increased protein levels of NGN3 coupled with the reduced transcript levels of NGN3 target genes (Fig. 3d), it seems that NGN3 does not effectively activate its target genes in P-KO EPs. We expect that either P-KO itself or a lack of arginine methylation suppresses the functionality of NGN3 in hESCs during pancreatic EC development.

Next, we investigated the developmental competence of P-KO EPs to become pancreatic ECs. We found some EC-associated genes (i.e., *NKX2.2* and *CHGA*) with reduced expression in P-KO ECs and others (i.e., *PDX1, HNF1b*, and *MAFA*) with normal expression (Fig. 3f). The transcript levels of genes downstream of NGN3 (i.e., *NEUROD1, PAX6*, and *PAX4*) were reduced in P-KO ECs (Fig. 3f and Supplementary Fig. 3h, i). Among the pancreatic endocrine hormone genes, we found specific reductions in insulin (*INS*) and somatostatin (*SST*) expression at the mRNA and protein levels in P-KO ECs (Fig. 3g, h). Pancreatic polypeptide (*PPY*) mRNA and protein expression levels were slightly but nonsignificantly increased in P-KO ECs (Fig. 3g, h). Our results imply that PRMT1 KO leads to reduced transcriptional activity of NGN3, thereby impairing the competence of EPs to become β or δ cells in vitro. To determine whether P-KO influences the subcellular localization of NGN3, we examined NGN3 in the cytoplasmic and nuclear compartments of P-iKO EPs. Compared to untreated cells, dox-treated P-iKO EPs showed higher NGN3 protein in whole-cell lysate (WCL) and in the cytoplasm (Cyt) but no difference in the nuclear (Nuc) compartment (Fig. 3i and Supplementary Figs. 4–5). Interestingly, slow migrating bands, presumably phosphorylated forms of NGN3, were observed in the Nuc fraction but not in the Cyt fraction (Fig. 3i). These data suggest that PRMT1-KO causes cytoplasmic NGN3 accumulation and leads to impaired fate choice of EPs.

### PRMT1 specifically methylates NGN3 arginine 65

**Fig. 4:**
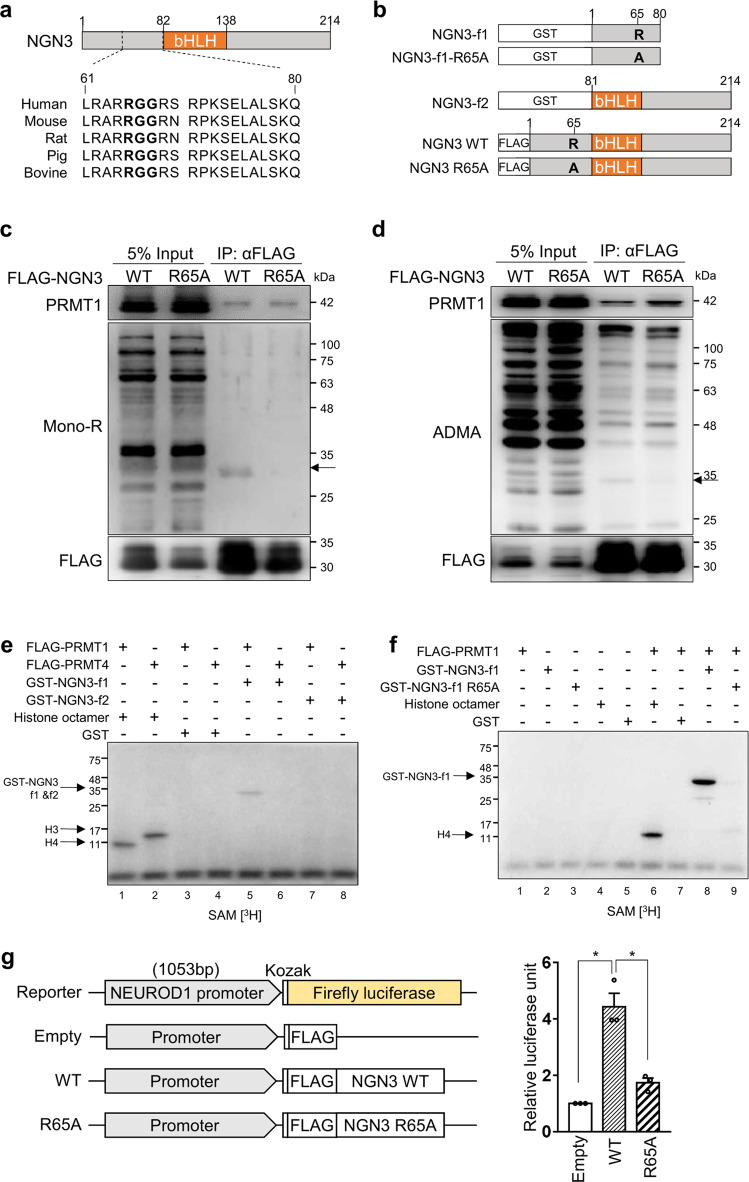
PRMT1-mediated methylation of NGN3 arginine 65. **a** An RGG motif (bold) in human NGN3 is conserved across species. bHLH, basic helix-loop-helix domain. **b** Several human NGN3 constructs for arginine methylation analysis. GST-tagged NGN3 fragments 1 (f1) and 2 (f2) were inserted into pGEX4T-1 for methyl transferase assays. FLAG-tagged NGN3 constructs were inserted into pCAG-FLAG-IPuro for NGN3 overexpression. Arginine 65 (R) of NGN3 was changed to alanine (A) to prevent arginine methylation. **c** Immunoprecipitation of FLAG-NGN3 with MG132 treatment. A mono-R band (arrow) was detected for FLAG-NGN3 WT but not for the FLAG-NGN3 mutant. mono-R, mono-methylated arginine. **d** Immunoprecipitation of FLAG-NGN3 without MG132 treatment. A di-methylated ADMA band was detected only with FLAG-NGN3 WT. ADMA, asymmetrical dimethylated arginine. **e**, **f** In vitro NGN3 methylation assays. NGN3 arginine 65 was methylated in the presence of [^3^H]SAM by PRMT1 (e, Lane 5). The R65A NGN3 mutant was not methylated in the presence of [^3^H]SAM by PRMT1 (f, Lane 8). Histone octamers were used to test FLAG-PRMT1 and FLAG-PRMT4 activities. The GST tag served as a negative control. See also Supplementary Fig. 6. **g** Luciferase assay for NGN3 activity in HEK cells using the *NEUROD1* promoter (1053 bp). The firefly luciferase signal for each experiment was normalized to the Renilla luciferase signal. The data are presented as the mean ± SEM. *p < 0.05 (*n* = 3). See also Supplementary Fig. 7.

PRMT1 preferentially methylates the arginine residues of RGG/RXR sequences in glycine- and arginine-rich (GAR) motifs in histone and nonhistone proteins^15,19,34^. According to UniProt (www.uniprot.org), NGN3 has a highly conserved RGG motif (amino acids 65–67) near its basic-helix-loop-helix (bHLH) domain (aa 83–135) (Fig. 4a). To determine whether arginine methylation of NGN3 is regulated by PRMT1, we first generated various FLAG-NGN3 expression vectors. These included vectors expressing intact NGN3, NGN3 fragment 1 (amino acids 1–80, f1), and NGN3 fragment 2 (amino acids 81–214, f2) (Fig. 4b). We also generated NGN3 R65A mutants by replacing arginine 65 (CGC) with alanine (GCC), expecting that PRMT1 would not be able to methylate this site. We then transfected FLAG-NGN3 WT or FLAG-NGN3 R65A mutant plasmids into HEK cells and performed immunoprecipitation followed by western blot analyses with methylated arginine-specific antibodies. We were able to detect mono- and dimethylated arginine bands at the position that corresponds to FLAG-NGN3 protein (arrows) only in the FLAG-NGN3 WT eluate but not in the FLAG-NGN3 R65A eluate (Fig. 4c, d). It is also worth noting that FLAG-NGN3 coimmunoprecipitated endogenous PRMT1 in an arginine 65-independent manner, implying a physical interaction between NGN3 and PRMT1. These results indicate that PRMT1 interacts with and methylates the arginine 65 residue of NGN3.

For in vitro methyltransferase assays, we purified FLAG-tagged recombinant PRMT family proteins and the GST-tagged NGN3 protein fragments f1 and f2 (Supplementary Fig. 6a). While both PRMT1 and PRMT4 exhibited intrinsic methyltransferase activity toward histone H4 and H3, respectively, only PRMT1, not PRMT4, could methylate NGN3-f1 (Fig. 4e). In addition, among the nine purified PRMT family proteins tested, only PRMT1 exhibited NGN3 methylation activity (Supplementary Fig. 6b), indicating that PRMT1 is the main arginine methyltransferase for NGN3, which produces mono- and dimethylation states (Supplementary Fig. 6c). In addition, PRMT1 was found to be unable to methylate NGN3-f2 (Fig. 4e), suggesting that PRMT1 methylates the N-terminal region of NGN3. In support of this and consistent with the above cellular analysis, purified R65A-NGN3-f1 was not methylated by PRMT1 (Fig. 4f). Taken together, our cellular and biochemical analyses confirm that PRMT1 specifically methylates NGN3 at arginine 65.

Next, we explored whether arginine methylation of NGN3 influences the expression of its downstream genes. After transfecting NGN3 WT and R65A into HEK cells, we found that the R65A mutant triggered less activation of *NEUROD1* than the WT (Supplementary Fig. 7). We then confirmed this reduced transcriptional activity of the R65A mutant via luciferase assay. To do so, we cotransfected HEK cells with each expression vector and a firefly luciferase reporter containing the *NEUROD1* promoter^32,35^. We first confirmed that the expression of each FLAG-tagged version of NGN3 was similar (Supplementary Fig. 7b) and then normalized the firefly luciferase signal to the Renilla luciferase signal. As expected, we observed significantly higher activity triggered by the NGN3 WT transfectants than by the R65A transfectants (Fig. 4g, *p* < 0.05). To determine the effects of the NGN3 R65A mutant on pancreatic development, H1 PEs were transfected with the NGN3 R65A mutant. Although PEs transfected with NGN3 WT and NGN3 R65A showed similar morphology, the NGN3 mutant group had a lower transcript level of the *NEUROD1* gene than the NGN3 WT group, not a lower transcript level of *PDX1* (a nontarget gene of NGN3) (Supplementary Fig. 8a). When the NGN3 mutant was transfected into P-iKO PEs, the transcriptional activity of *NEUROD1* and *PAX6* was reduced in the dox(+) P-iKO PEs compared to the dox(−) P-iKO PEs (Supplementary Fig. 8b). These results indicate that the R65A mutation diminishes NGN3 transcriptional activity. Together, our findings suggest that arginine 65 methylation of NGN3 is essential for transcriptional activity.

### Arginine 65 methylation of NGN3 is a prerequisite for its degradation

**Fig. 5:**
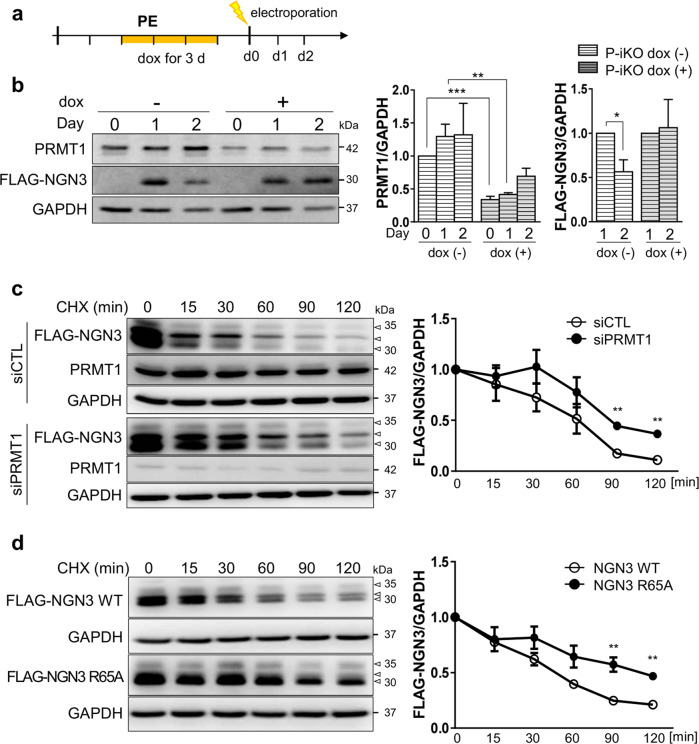

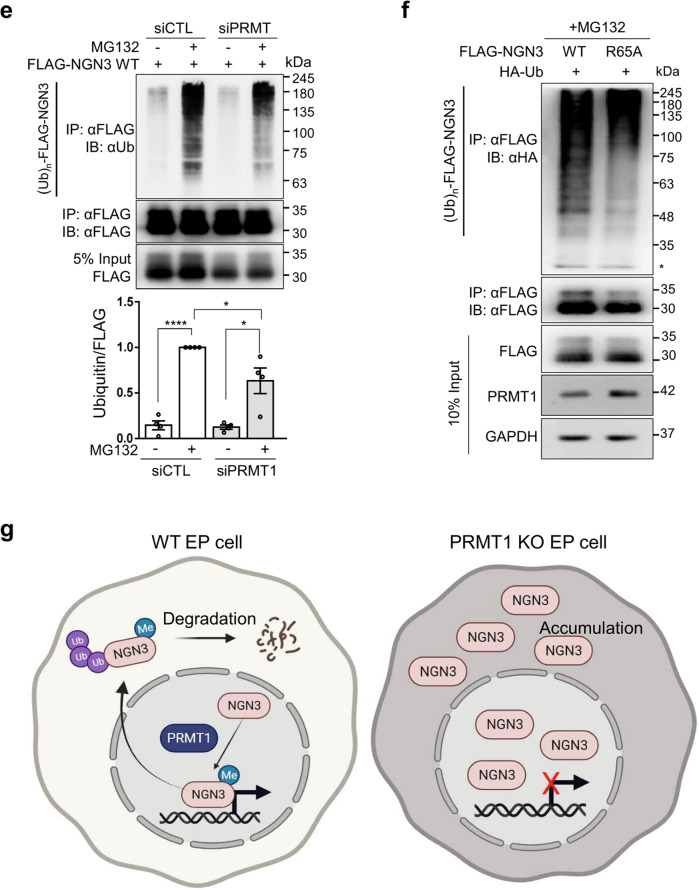
Degradation of arginine 65-methylated NGN3. **a** Protocol for the treatment of P-iKO PEs with dox and electroporation with the FLAG-NGN3 expression vector. **b** Accumulation of NGN3 protein in P-KO PEs. P-iKO PEs treated with dox(+) showed downregulation of PRMT1 but not FLAG-NGN3. The data are presented as the mean ± SEM. *p < 0.05, **p < 0.01, ***p < 0.001 (*n* = 4). **c** Degradation assay for FLAG-NGN3 in HEK cells treated with PRMT1-siRNA. In the presence of CHX, we observed delayed degradation of FLAG-NGN3 in PRMT1-KD HEK cells. FLAG-NGN3 proteins appear as multiple bands (arrowheads). FLAG-NGN3 levels in cells treated with siPRMT1 compared to those treated with siCTL at the indicated time points after CHX treatment. The data are presented as the mean ± SEM. **p < 0.01 (*n* = 3). CHX, cycloheximide; siCTL, siRNA control (unspecific sequences). **d** Delayed degradation of the FLAG-NGN3 R65A mutant in HEK cells. The FLAG-NGN3 R65A mutant was degraded slower than FLAG-NGN3 WT. FLAG-NGN3 R65A mutant levels compared to FLAG-NGN3 WT at the indicated time points after CHX treatment. The data are presented as the mean ± SEM. **p < 0.01 (*n* = 3). See also Supplementary Fig. 9. **e** Reduced polyubiquitination of FLAG-NGN3 in PRMT1-KD HEK cells. HEK cells transfected with PRMT1-siRNA showed reduced ubiquitination. The intensity of the (Ub)n-FLAG-NGN3 area was normalized by the αFLAG-NGN3 IP band. Ub, ubiquitin. *p < 0.05, ****p < 0.0001 (*n* = 4). **f** Decreased polyubiquitination of the FLAG-NGN3 R65A mutant in HEK cells. **g** Putative model for the role of arginine 65 methylation of NGN3 in pancreatic EPs. Arginine-methylated NGN3 activates NGN3 target genes such as *NEUROD1* in the nucleus and is then rapidly degraded by ubiquitination at the pancreatic EP stage. PRMT1-KO prevents NGN3 arginine methylation, leading to accumulation of NGN3 and reduced activation of its target genes. Ultimately, defective NGN3 arginine 65 methylation prevents the cell fate transition of EPs to ECs. The diagram was generated using BioRender (biorender.com).

NGN3 is transiently expressed in pancreatic EPs, rapidly disappearing during pancreatic lineage development^12^. Therefore, we investigated whether NGN3 arginine methylation is associated with degradation. To answer this question, we transfected P-iKO PEs with an NGN3 expression vector and incubated them for 1–2 d (Fig. 5a). Although ectopic NGN3 expression was significantly reduced 2 days after transfection in P-iKO PEs that were not treated with dox, similar cells treated with dox maintained consistent levels of NGN3 (Fig. 5b). This suggests that PRMT1 is associated with NGN3 degradation in hESCs during pancreatic differentiation.

To investigate the role of PRMT1 in NGN3 degradation, we transfected HEK cells with PRMT1-specific siRNAs, transfected them with a FLAG-NGN3 expression vector, and then treated them with cycloheximide (CHX) to block protein synthesis. We observed a significant delay in FLAG-NGN3 degradation in the siPRMT1-treated cells compared to the siCTL-treated cells (Fig. 5c, *p* < 0.01). We also found that FLAG-NGN3 R65A showed a longer half-life than WT (Fig. 5d, *p* < 0.01). Unlike R65A-NGN3, WT-NGN3 appeared in multiple distinct bands on the western blot (Fig. 5d). In addition, we observed differences in the phosphorylation bands between the FLAG-NGN3 WT and R65A (Supplementary Fig. 9a, arrowheads) that were sensitive to phosphatase treatment (Supplementary Fig. 9b, arrowheads). These results indicate that arginine methylation contributes to NGN3 stability by altering NGN3 phosphorylation. However, phospho-serine-specific antibodies for NGN3 are not yet commercially available, and the exact phospho-serine site influenced by arginine 65 methylation remains elusive.

To examine the correlation between arginine methylation and polyubiquitination of NGN3, we transfected HEK cells with FLAG-NGN3 and siPRMT1 and then treated them with the proteasome inhibitor MG132 (20 μM) for 2 h. In the presence of MG132, the ubiquitinated NGN3 protein significantly accumulated in both groups, but its accumulated level was significantly reduced in the siPRMT1 group compared to the siCTL group (Fig. 5e). R65A-NGN3 also showed weaker ubiquitination bands than WT-NGN3 (Fig. 5f). These results indicate that arginine 65 methylation of NGN3 by PRMT1 is critical for NGN3 degradation in the process of EC development.

## Discussion

Here, we report for the first time that arginine 65 methylation of NGN3 is critical for the pancreatic lineage differentiation of hESC-derived EPs into ECs. While we did find that P-KO EPs exhibited increased NGN3 protein expression, they did not develop into pancreatic ECs in vitro. Using an in vitro methylation assay, we found that PRMT1 specifically methylates arginine 65 on the GAR motif of NGN3. Preventing methylation blocked ubiquitin-mediated NGN3 degradation and activity. Thus, we found that PRMT1 is a key regulator of NGN3 function in pancreatic development via direct PRMT1-NGN3 crosstalk.

Arginine methylation of nonhistone proteins is important in several cellular functions, such as transcription, EMT, and cellular proliferation^17–20,25^. However, the role of PRMT1-mediated arginine methylation in pancreatic development is poorly understood. P-KO mouse embryos exhibit prolonged NGN3 expression accompanied by reduced pancreatic β-cell mass and pancreatic hypoplasia after birth^26^. This suggests that PRMT1 may control NGN3 stability in mammalian pancreas development. Transient expression or rapid degradation of NGN3 is necessary for cell fate determination of EPs into ECs^8,11,36,37^. To study the molecular mechanism underlying the role of PRMT1 in NGN3 stability as it relates to pancreatic development, we generated a P-iKO hESC line and guided its differentiation into the pancreatic endocrine lineage. Despite elevated levels of NGN3 protein, P-KO EPs did not develop into ECs (Fig. 3). Furthermore, P-KO EPs and ECs exhibited reductions in the expression of NGN3 downstream genes (i.e., *NEUROD1, PAX6, PAX4, NKX2.2*, and *CHGA*). These results suggest that P-KO EPs have NGN3 dysfunction. PRMT1 deficiency affects fate decisions in specialized ECs, as P-KO ECs show significantly reduced expression of β- and δ-cell markers. Since β- and δ- cells arise from the same ancestor^38,39^, it is clear that PRMT1 contributes to the specification of EPs into β- and δ- cells. However, GCG+ cells were not detected from this differentiation, so further investigation is required to reveal the role of PRMT1 in pancreatic α-cell specification. We found that arginine 65 of NGN3 is a conserved methylation motif among mammals whose methylation is specifically catalyzed by PRMT1. Furthermore, unmethylated NGN3 was not degraded via ubiquitin-mediated proteolysis. Our results indicate that NGN3 arginine 65 methylation is crucial for EC fate determination in pancreatic lineage development.

To induce PRMT1 loss of function at a specific stage of pancreatic development, we generated a PRMT1-inducible KO (P-iKO) system in hESCs using a “safe harbor” *AAVS1* locus. We selected this *AAVS1* locus because it does not produce any adverse effects on differentiation, transgene silencing, or proliferation in gene-edited hiPSCs^40,41^. We performed homology-directed repair (HDR) using ZFNs to avoid random transgene integration. When we cultured P-iKO hESCs in the presence of dox, most cells died within 2 d. This implies that PRMT1 is essential for the viability and proliferation of hESCs, which is consistent with the embryonic lethal phenotype of PRMT1-KO mice^42^. In the absence of dox, however, P-iKO hESCs maintained pluripotency, differentiation competence, and Cas9 mRNA expression for more than 3 months. Thus, our use of the *AAVS1* locus with these transgenes permitted sufficiently stable gene expression for the maintenance and differentiation of hESCs.

The NGN3 protein is rapidly degraded by the canonical ubiquitination pathway^43^. In the mouse EPs, NGN3 is degraded by the ubiquitin‒proteasome system after phosphorylation^14,43^. Likewise, multiple phosphorylated NGN3 proteins were detected only in the nuclear fraction, not the cytoplasmic fraction, of hESC-derived EPs. We observed different electrophoretic mobility for the NGN3 R65A mutant than for WT NGN3, suggesting that arginine 65 methylation of NGN3 affects NGN3 phosphorylation. In addition, we found that most of the endogenous NGN3 protein in P-KO EPs was localized to the cytoplasm. As with the NGN3 R65A mutation, KD of PRMT1 slowed NGN3 degradation in HEK cells. Therefore, we conclude that arginine 65 methylation of NGN3 is associated with NGN3 phosphorylation, leading to NGN3 degradation. This degradation is required for the cell fate transition of EPs to ECs during human pancreatic lineage development.

From these results, we propose a model for the role of PRMT1 in pancreatic endocrine lineage development (Fig. 5g). In this model, NGN3 arginine 65 is specifically methylated by PRMT1. This permits the expression of NGN3 downstream genes such as *NEUROD1*, which is important for the transition of EPs to ECs. The methylation of NGN3 arginine 65 also induces ubiquitin-dependent degradation of NGN3 by the proteasome, permitting the completion of EC development. In the absence of PRMT1, NGN3 does not activate the expression of its downstream target genes, nor is it degraded to permit the EP-to-EC transition. Therefore, we conclude that arginine 65 methylation of NGN3 is critical for EC fate determination during pancreatic development.

## Supplementary information

Supplementary information

## References

[1] Levetan C (2010). Distinctions between islet neogenesis and β-cell replication: implications for reversal of Type 1 and 2 diabetes. J. Diabetes.

[2] D’Amour KA (2005). Efficient differentiation of human embryonic stem cells to definitive endoderm. Nat. Biotechnol..

[3] Pan FC, Brissova M (2014). Pancreas development in humans. Curr. Opin. Endocrinol. Diabetes Obes..

[4] Offield MF (1996). PDX-1 is required for pancreatic outgrowth and differentiation of the rostral duodenum. Development.

[5] Bouwens L, Klöppel G (1996). Islet cell neogenesis in the pancreas. Virchows Arch..

[6] Schwitzgebel VM (2000). Expression of neurogenin3 reveals an islet cell precursor population in the pancreas. Development.

[7] Jennings RE, Scharfmann R, Staels W (2020). Transcription factors that shape the mammalian pancreas. Diabetologia.

[8] Smith SB, Watada H, German MS (2004). Neurogenin3 activates the islet differentiation program while repressing its own expression. Mol. Endocrinol..

[9] Gu G, Dubauskaite J, Melton DA (2002). Direct evidence for the pancreatic lineage: NGN3+ cells are islet progenitors and are distinct from duct progenitors. Development.

[10] Jennings RE, Berry AA, Strutt JP, Gerrard DT, Hanley NA (2015). Human pancreas development. Development.

[11] Gradwohl G, Dierich A, LeMeur M, Guillemot F (2000). Neurogenin3 is required for the development of the four endocrine cell lineages of the pancreas. Proc. Natl Acad. Sci. USA.

[12] Apelqvist Å (1999). Notch signalling controls pancreatic cell differentiation. Nature.

[13] Azzarelli R (2017). Multi-site Neurogenin3 phosphorylation controls pancreatic endocrine differentiation. Dev. Cell.

[14] Krentz NAJ (2017). Phosphorylation of NEUROG3 links endocrine differentiation to the cell cycle in pancreatic progenitors graphical abstract HHS public access. Dev. Cell.

[15] Bedford MT, Richard S (2005). Arginine methylation: an emerging regulator of protein function. Mol. Cell.

[16] Tang J (2000). PRMT1 is the predominant type I protein arginine methyltransferase in mammalian cells. Jbc.

[17] Avasarala S (2015). PRMT1 Is a novel regulator of epithelial-mesenchymal-transition in non-small cell lung cancer. J. Biol. Chem..

[18] Bedford MT, Clarke SG (2009). Protein arginine methylation in mammals: who, what, and why. Mol. Cell.

[19] Gary JD, Clarke S (1998). RNA and protein interactions modulated by protein arginine methylation. Prog. Nucleic Acid Res. Mol. Biol..

[20] Raposo AE, Piller SC (2018). Protein arginine methylation: an emerging regulator of the cell cycle. Cell Div..

[21] Wang H (2001). Methylation of histone H4 at arginine 3 facilitating transcriptional activation by nuclear hormone receptor. Science.

[22] Strahl BD (2001). Methylation of histone H4 at arginine 3 occurs in vivo and is mediated by the nuclear receptor coactivator PRMT1. Curr. Biol..

[23] Gen S (2020). Stability of tuberous sclerosis complex 2 is controlled by methylation at R1457 and R1459. Sci. Rep..

[24] Malbeteau L (2020). PRMT1 is critical for the transcriptional activity and the stability of the progesterone receptor. iScience.

[25] Yamagata K (2008). Arginine methylation of FOXO transcription factors inhibits their phosphorylation by Akt. Mol. Cell.

[26] Lee K (2019). Essential role of protein arginine methyltransferase 1 in pancreas development by regulating protein stability of neurogenin 3. Diabetes Metab. J..

[27] Bertero A (2016). Optimized inducible shRNA and CRISPR/Cas9 platforms for in vitro studies of human development using hPSCs. Dev.

[28] Snijders, K. E., Cooper, J. D., Vallier, L. & Bertero, A. Conditional gene knockout in human cells with inducible CRISPR/Cas9. *Cris. Gene Ed*. **1961**, 185–209 (2019). 10.1007/978-1-4939-9170-9_12 30912047

[29] Kim Y (2016). Islet-like organoids derived from human pluripotent stem cells efficiently function in the glucose responsiveness in vitro and in vivo. Sci. Rep..

[30] Kim J, Roeder RG (2011). Nucleosomal H2B ubiquitylation with purified factors. Methods.

[31] Roark R, Itzhaki L, Philpott A (2012). Complex regulation controls Neurogenin3 proteolysis. Biol. Open.

[32] Huang H-P (2000). Regulation of the Pancreatic Islet-Specific GeneBETA2 (neuroD) by Neurogenin 3. Mol. Cell. Biol..

[33] Sander M (1997). Genetic analysis reveals that PAX6 is required for normal transcription of pancreatic hormone genes and islet development. Genes Dev..

[34] Thandapani P, O’Connor TR, Bailey TL, Richard S (2013). Defining the RGG/RG Motif. Mol. Cell.

[35] Wang J (2006). Mutant Neurogenin-3 in congenital malabsorptive diarrhea. N. Engl. J. Med..

[36] Miyatsuka T, Kosaka Y, Kim H, German MS (2011). Neurogenin3 inhibits proliferation in endocrine progenitors by inducing Cdkn1a. Proc. Natl Acad. Sci. USA.

[37] Salisbury, R. J. et al. The window period of NEUROGENIN3 during human gestation. *Islets* **6**, e954436 (2014). 10.4161/19382014.2014.954436 PMC4376053 25322831

[38] DiGruccio MR (2016). Comprehensive alpha, beta and delta cell transcriptomes reveal that ghrelin selectively activates delta cells and promotes somatostatin release from pancreatic islets. Mol. Metab..

[39] Mfopou JK, Chen B, Sui L, Sermon K, Bouwens L (2010). Recent advances and prospects in the differentiation of pancreatic cells from human embryonic stem cells. Diabetes.

[40] Hockemeyer D (2011). Genetic engineering of human pluripotent cells using TALE nucleases. Nat. Biotechnol..

[41] Smith JR (2008). Robust, persistent transgene expression in human embryonic stem cells is achieved with AAVS1-targeted integration. Stem Cells.

[42] Pawlak MR, Scherer CA, Chen J, Roshon MJ, Ruley HE (2000). Arginine N-methyltransferase 1 is required for early postimplantation mouse development, but cells deficient in the enzyme are viable. Mol. Cell. Biol..

[43] Vosper JMD (2009). Ubiquitylation on canonical and non-canonical sites targets the transciption factor neurogenin for ubiquitin-mediated proteolysis. J. Biol. Chem..

