# Supplementary material for: Arginine 65 methylation of Neurogenin 3 by PRMT1 is required for pancreatic endocrine development of hESCs
Source: Exp Mol Med. 2023 Jul 3;55(7):1506–19. doi: 10.1038/s12276-023-01035-8 (PMC10393949; doi:10.1038/s12276-023-01035-8)
Supplement: Supplementary file 1 — Supplementary information [file 12276_2023_1035_MOESM1_ESM.pdf]

# Supplementary Fig. 1

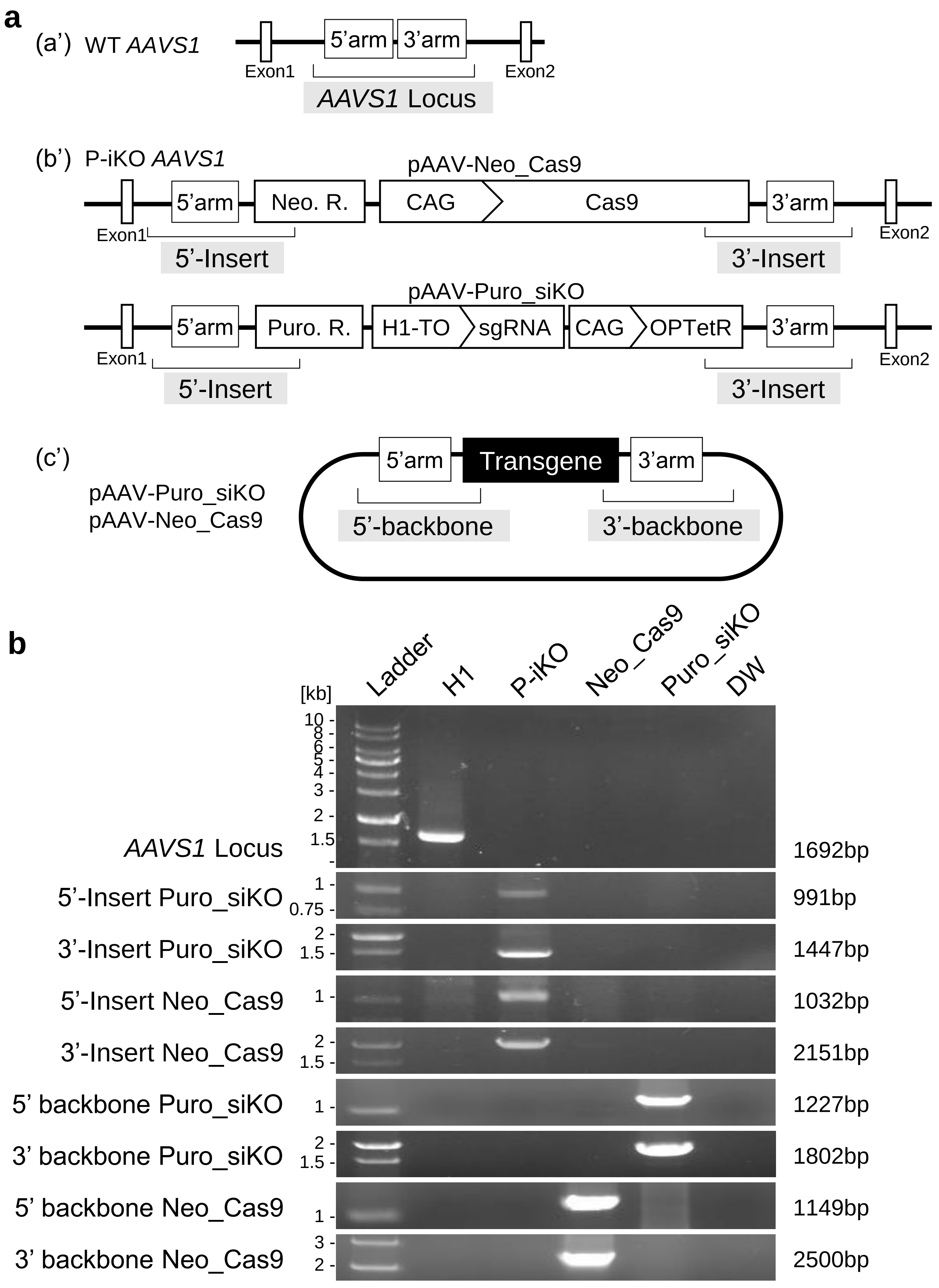

**Supplementary Fig. 1 Genotyping of P-iKO hESCs, *related to Fig. 1***

(a) Schematic P-iKO constructs for targeting the AAVS1 genomic locus. AAVS1 genomic locus of H1 hESCs (a’, WT AAVS1), both P-iKO hESC targeted AAVS1 alleles (b’, P-iKO AAVS1), and the AAVS1 targeting vector (c’, pAAV-Puro_siKO, pAAV-Neo_Cas9). Neo. R., Neomycin resistance gene; Puro. R., Puromycin resistance gene; H1-TO, tetracycline-inducible H1 RNA Polymerase III promoter containing a tetO2 sequence; OPTetR, OPTimized Tetracycline-responsive Repressor [1].

(b) Genotyping a P-iKO hESC line. DW was loaded as a negative PCR control.

# Supplementary Fig. 2

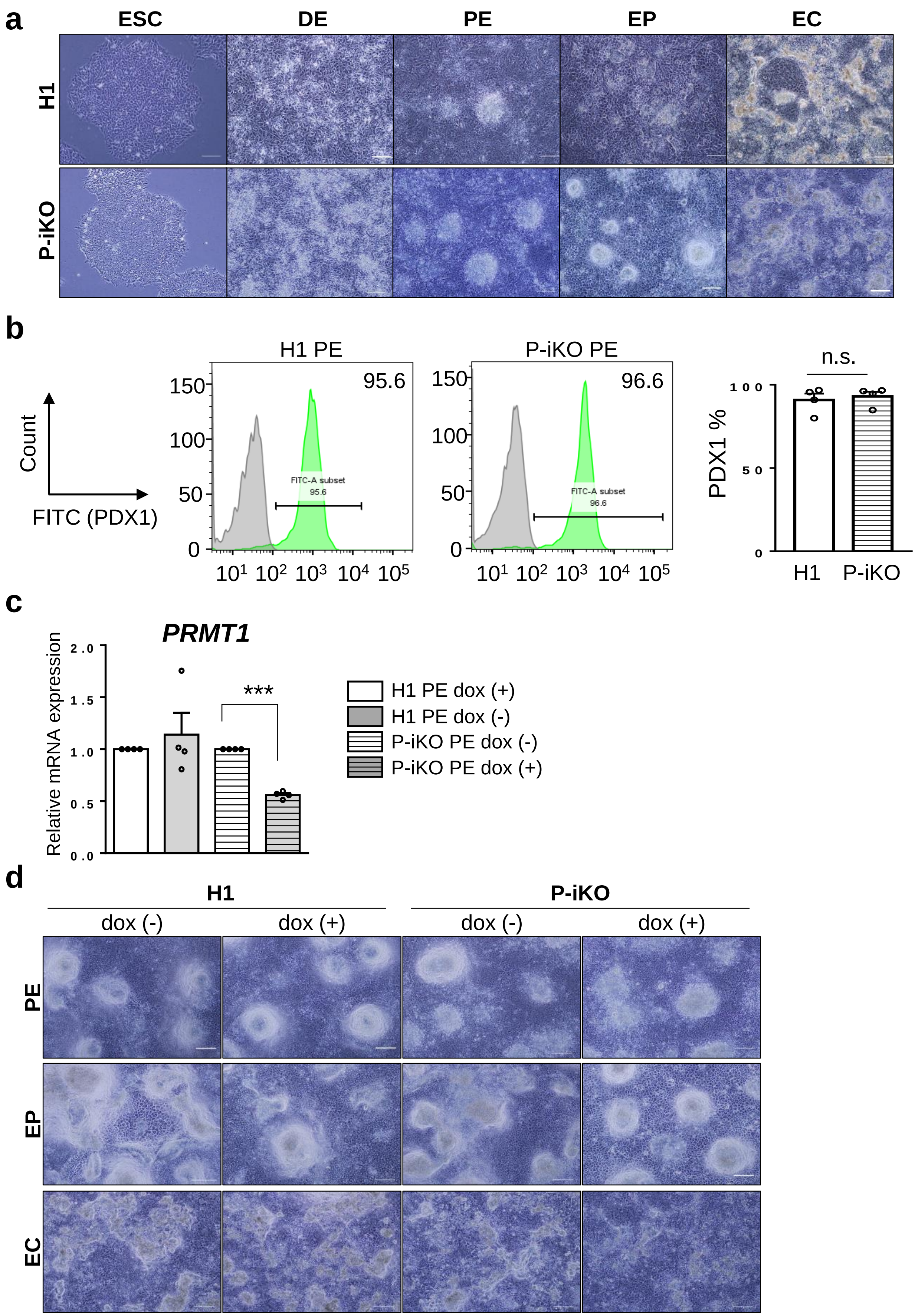

# Supplementary Fig. 2

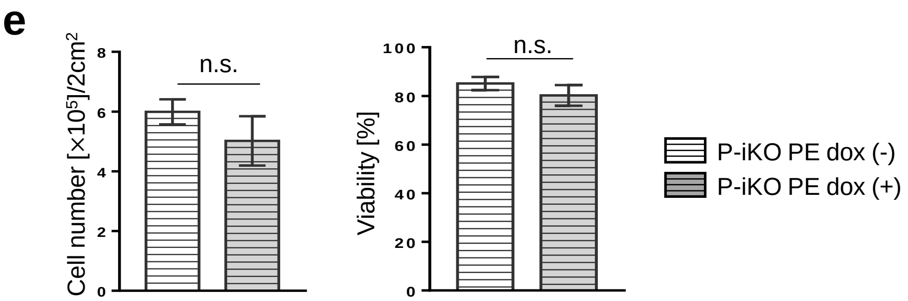


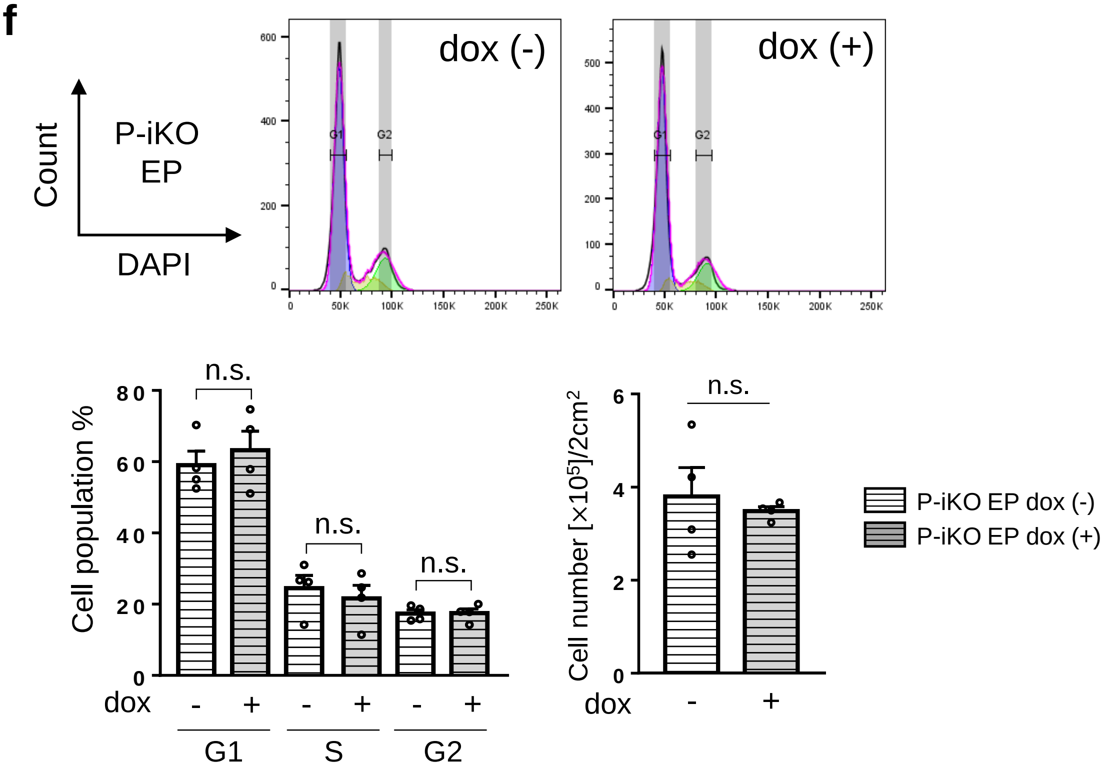


**Supplementary Fig. 2 Differentiation of P-iKO hESCs into pancreatic ECs, *related to Fig. 2***

(a) Cellular morphology at the various stages through which P-iKO hESCs progress as they differentiate into ECs. Scale bars, 200 μm.

(b) PDX1-positive population of H1 and P-iKO PEs without dox treatment. n.s., not significant. Data are represented as mean ± SEM (n = 4).

(c) Downregulation of *PRMT1* mRNA in P-KO PEs. Suppression of *PRMT1* was observed in dox (+) P-iKO PE cells on PE day 6 (PED6). ***$p < 0.001$. Data are represented as mean ± SEM (n = 4).

(d) Bright-field images for H1 and P-iKO PE, EP, and EC stages with and without dox treatment. Scale bars, 200 μm.

(e) Cell number and cell viability of P-iKO PEs by trypan blue staining. n.s., not significant. Data are represented as mean ± SEM (n = 4).

(f) Cell cycle analysis of P-iKO EPs. DNA contents of P-iKO EPs were analyzed by Flow cytometry with DAPI staining in both dox (-) and (+) condition. (n = 4), n.s., not significant. Cell cycle analysis was done by FlowJo Cell Cycle analysis function, Watson model used (v10.8).

# Supplementary Fig. 3

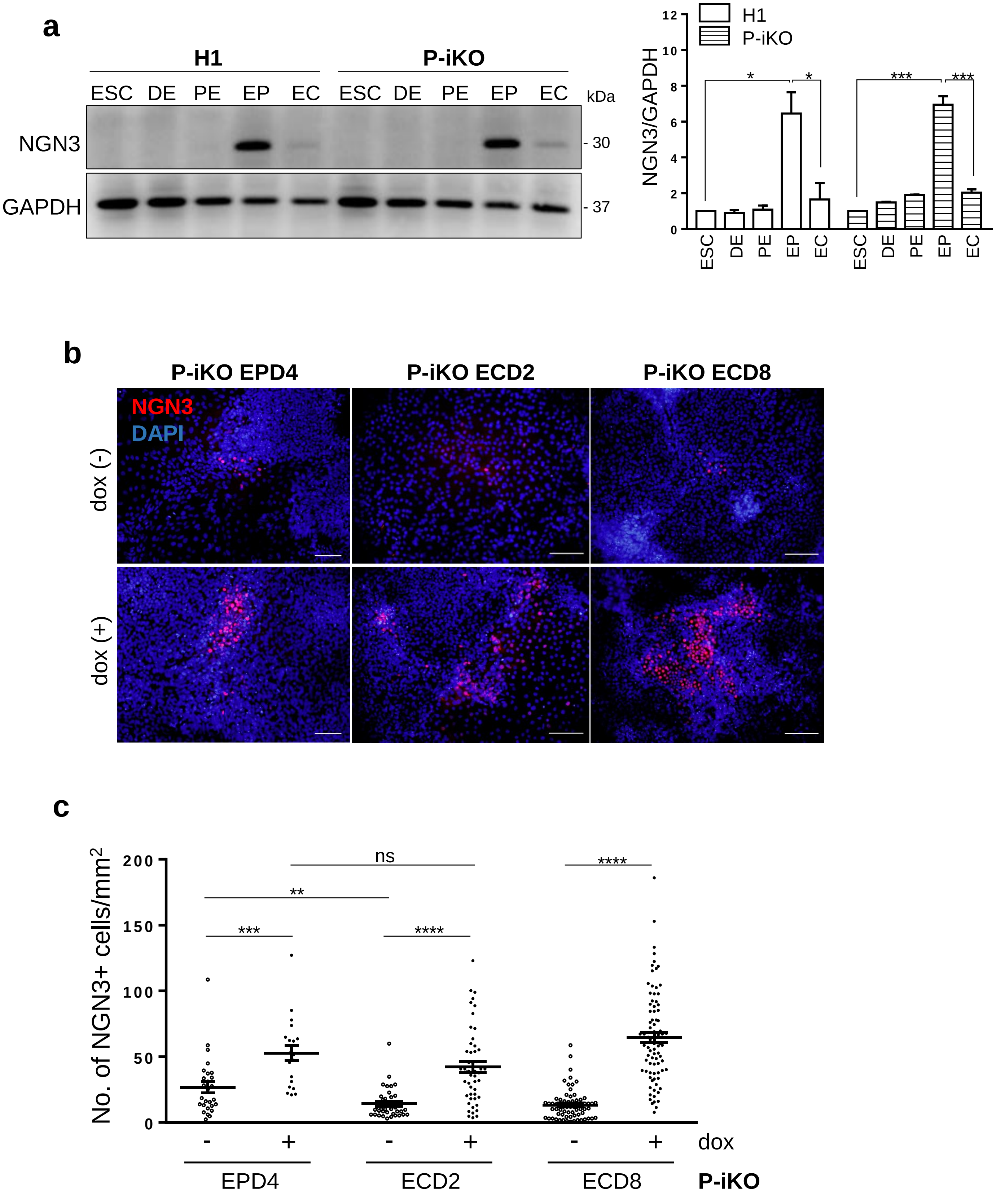

# Supplementary Fig. 3

**d**

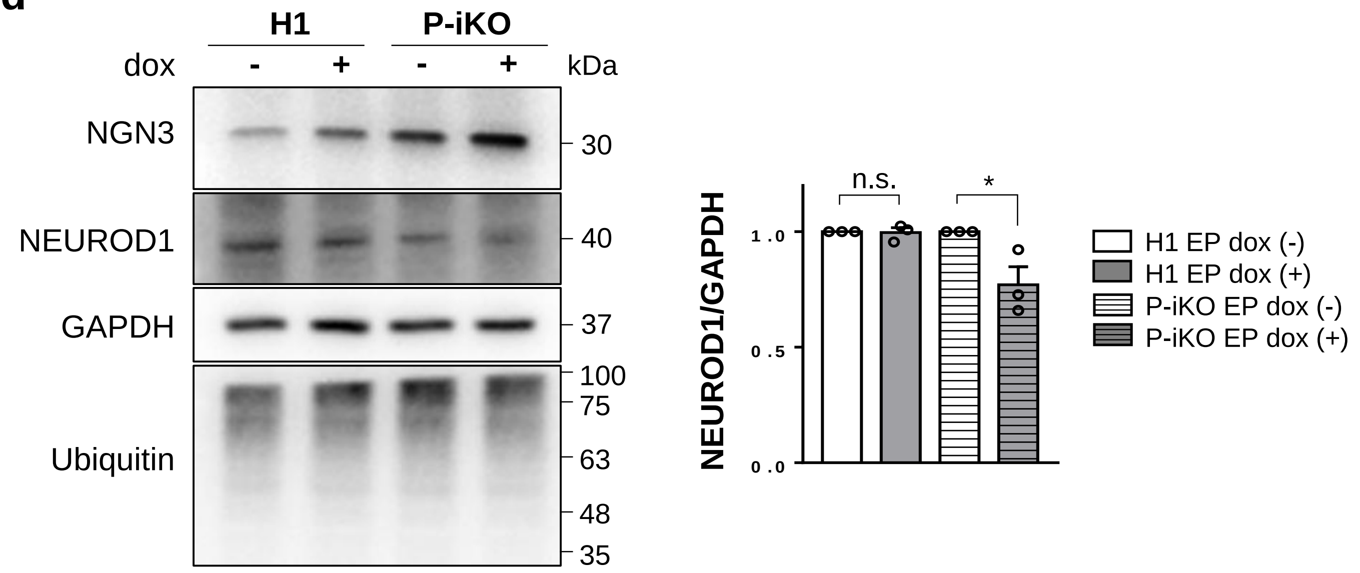


**e**

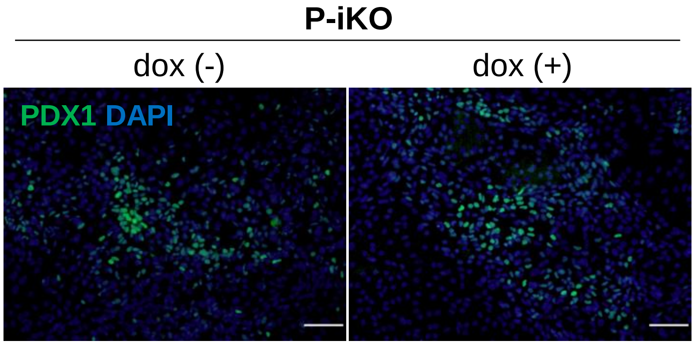


**f**

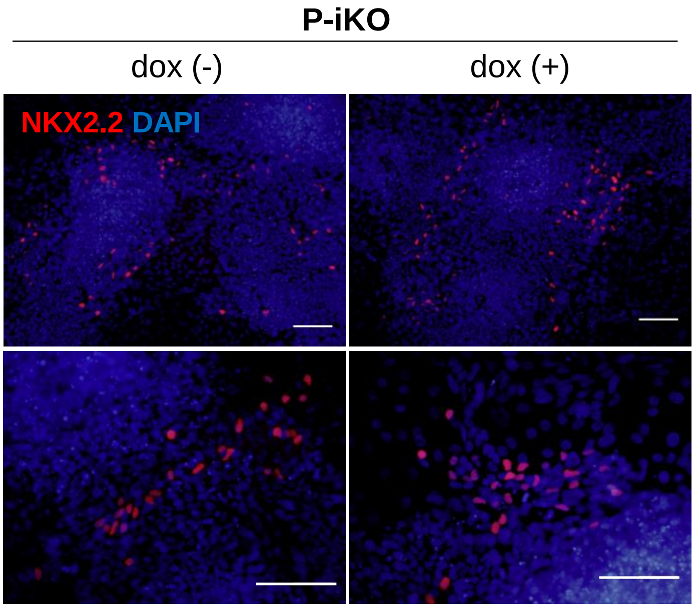


**g**

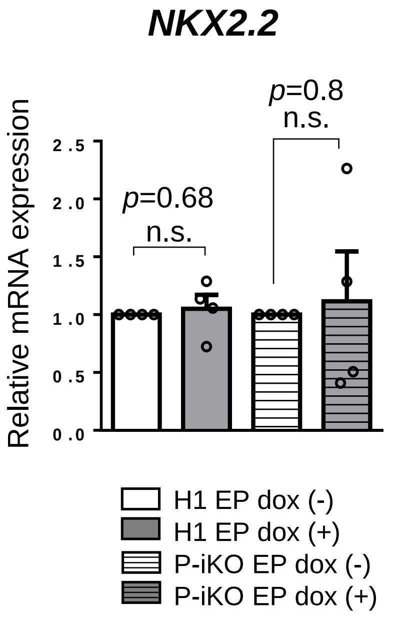

# Supplementary Fig. 3

h

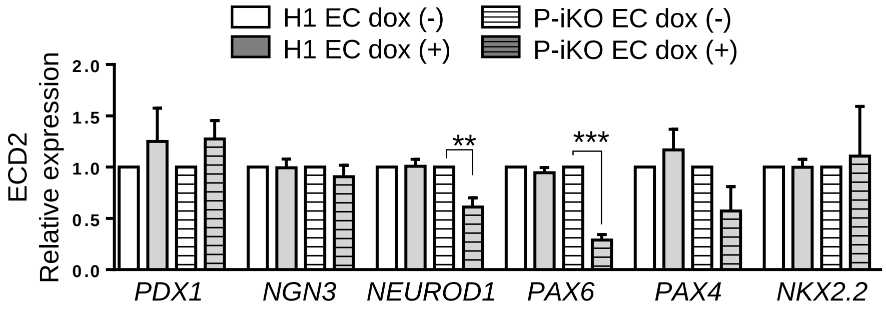


i

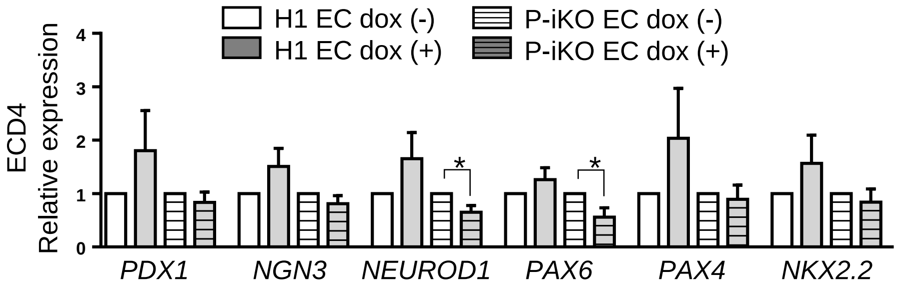


**Supplementary Fig. 3 Effects of PRMT1 depletion in P-KO EPs and ECs, *related to Fig. 3***

(a) Expression of NGN3 in P-iKO hESCs as they progress through pancreatic EC development. Transient NGN3 expression was detected only in EP cells derived from both H1 and P-iKO hESCs. *$p < 0.05$, ***$p < 0.001$. Data are represented as mean ± SEM (n = 3).

(b) More NGN3-positive cells among P-KO EPs and ECs on EP day 4 (EPD4), EC day 2 (ECD2), and EC day 8 (ECD8). Scale bars, 100 µm.

(c) More NGN3-positive cells among P-KO EPs and ECs. **$p < 0.01$, ***$p < 0.001$, ****$p < 0.0001$ (n > 20).

(d) Enhanced NGN3 and reduced NEUROD1 protein expression in dox (+) P-iKO EPs. Total ubiquitination level were not altered by PRMT1 KO in P-iKO EPs. Data are represented as mean ± SEM (n = 4).

(e) Immunostaining of PDX1 in P-iKO EPs. Scale bars, 100 µm.

(f) Immunostaining of NKX2.2 in P-iKO EPs. Scale bars, 100 µm.

(g) Relative mRNA expression of NKX2.2 in P-iKO EPs. n.s., not significant. Data are represented as mean ± SEM (n = 4).

(h) Reduced mRNA expression of the NGN3 target genes *NEUROD1* and *PAX6* in P-KO ECs on EC day 2 (ECD2). **$p < 0.01$, ***$p < 0.001$. Data are represented as mean ± SEM (n = 4).

(i) Significantly reduced mRNA expression of *NEUROD1* and *PAX6* in P-KO ECs on EC day 4 (ECD4). *$p < 0.05$. Data are represented as mean ± SEM (n = 4).

# Supplementary Fig. 4

**a**

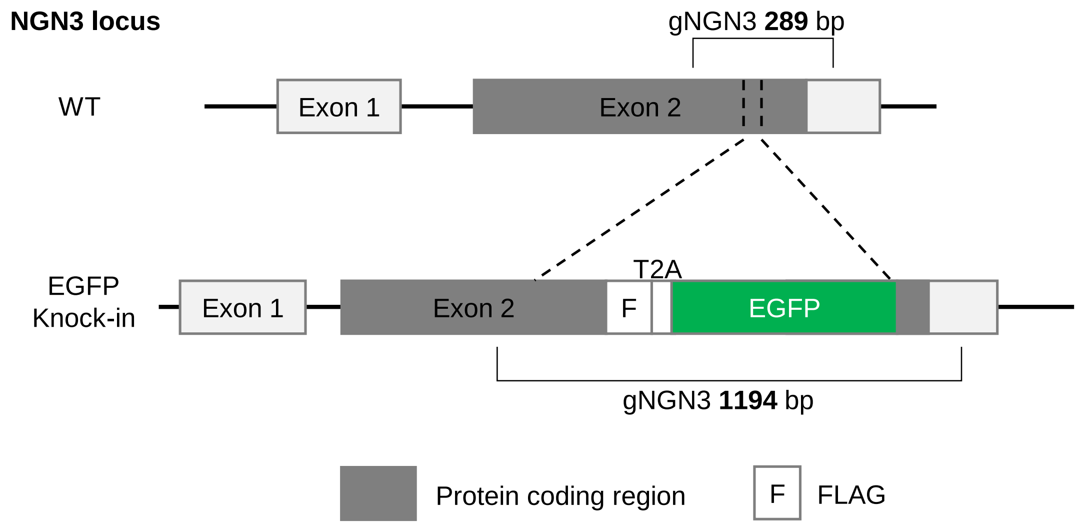


**b**

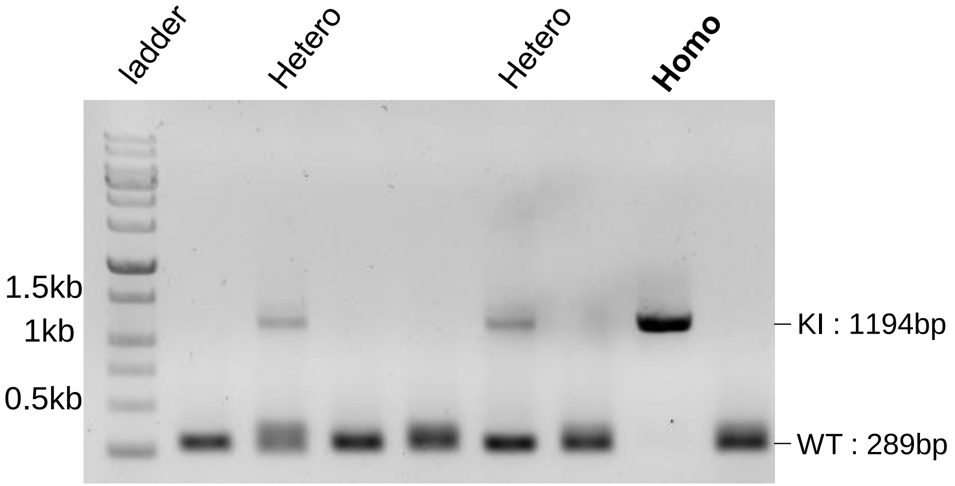


HDR efficiency: Hetero: 5/42 colonies (11.9%)
Homo: 1/42 colonies (2.38%)

**c**

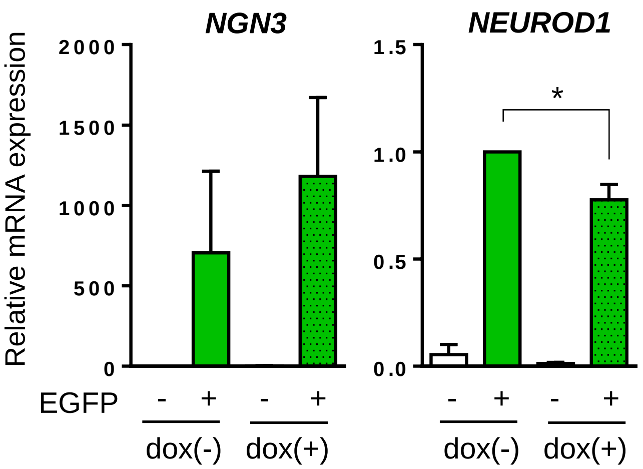

# Supplementary Fig. 4

d

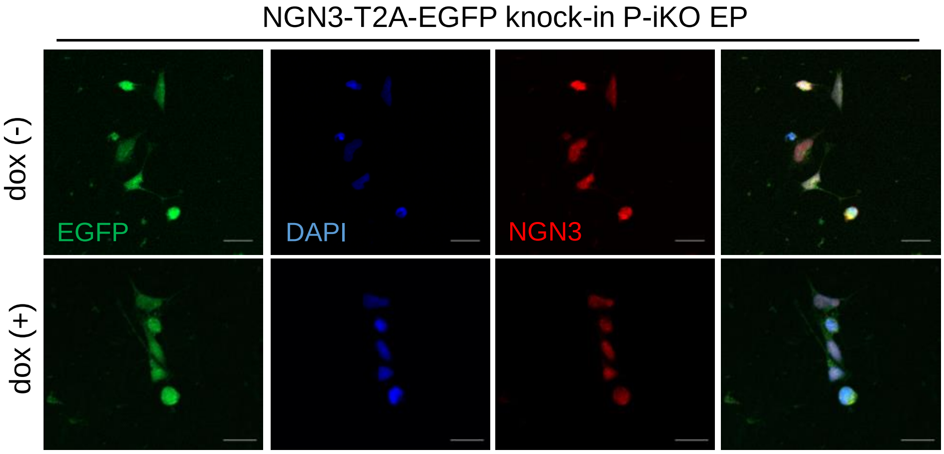


e

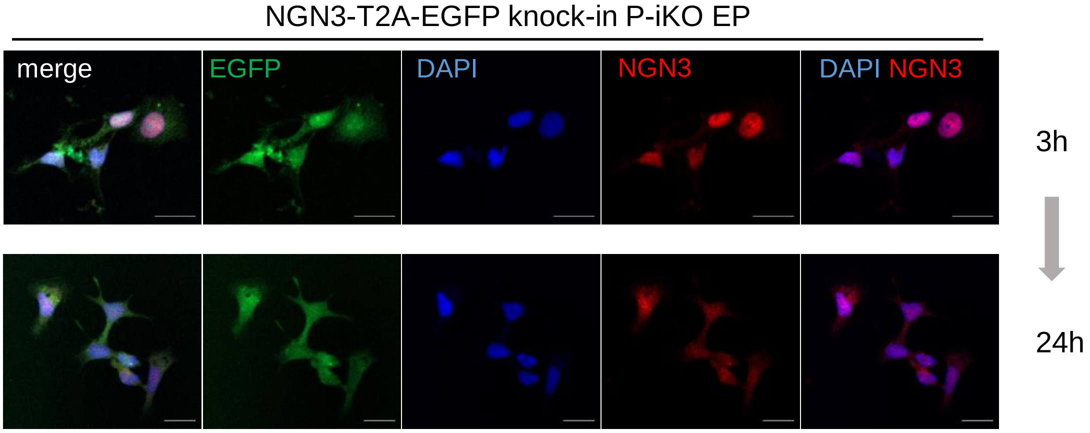


**Supplementary Fig. 4 NEUROD1 mRNA expression in NGN3-EGFP FACS sorted EPs, *related to Fig. 3***

(a) Schematic description of NGN3 genomic locus which targeted with EGFP reporter.
(b) Genotyping of NGN3-EGFP reporter construct. KI; knock-in.
(c) NGN3-T2A-EGFP knock-in P-iKO hESC-derived EPs were sorted by EGFP intensity. EGFP sorted P-iKO EPs showed reduced NEUROD1 mRNA expression in dox (+) compared to dox (-). **p* < 0.05, (n = 3)
(d) EGFP-sorted EPs clearly co-localized with NGN3 protein in both dox (-) and (+) EPs. Scale bars, 20 μm.
(e) NGN3 localization switching. NGN3 protein preliminary locates in the nucleus (upper panel) after 3 hrs of EGFP sorting at EPD4, and gradually dispersed into the cytoplasm (lower panel) after 24 hrs of EGFP sorting. Scale bars, 20 μm.

# Supplementary Fig. 5

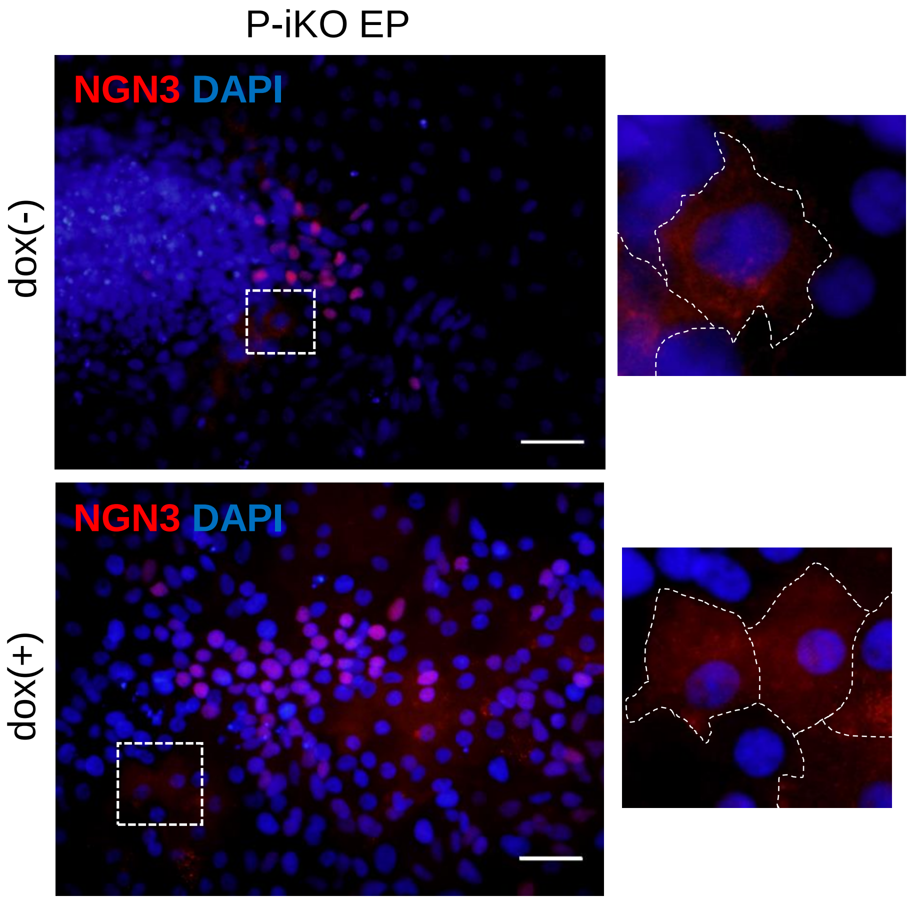


**Supplementary Fig. 5 Immunostaining of NGN3 of P-iKO EPs, *related to Fig. 3***

NGN3 immunostaining of P-iKO EPs (left) and magnified images (right). NGN3 mainly observed in the nucleus (left), however, both of the nuclear and cytoplasmic expression were detected (right, dotted-line). Scale bars, 50 μm.

# Supplementary Fig. 6

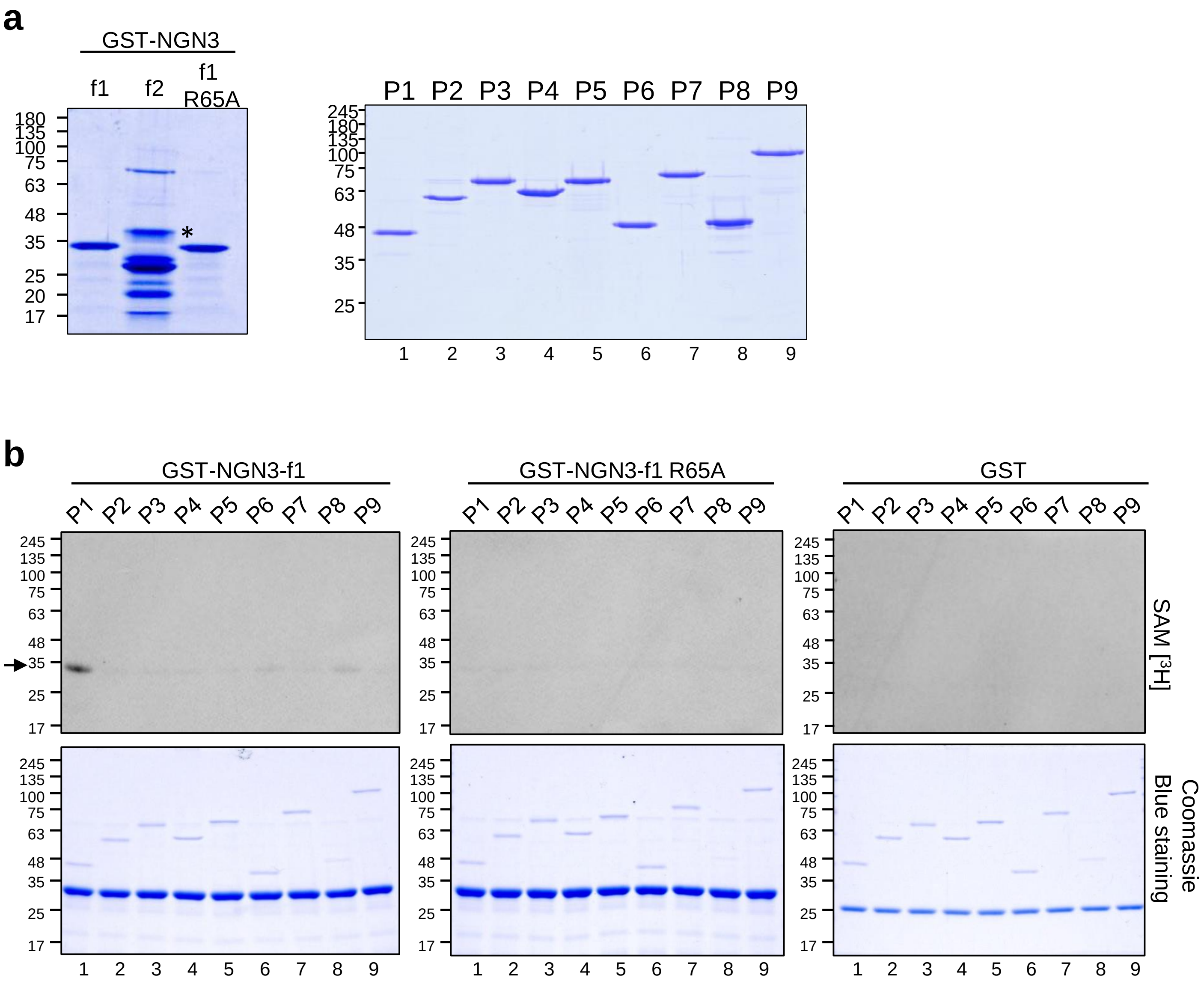

# Supplementary Fig. 6

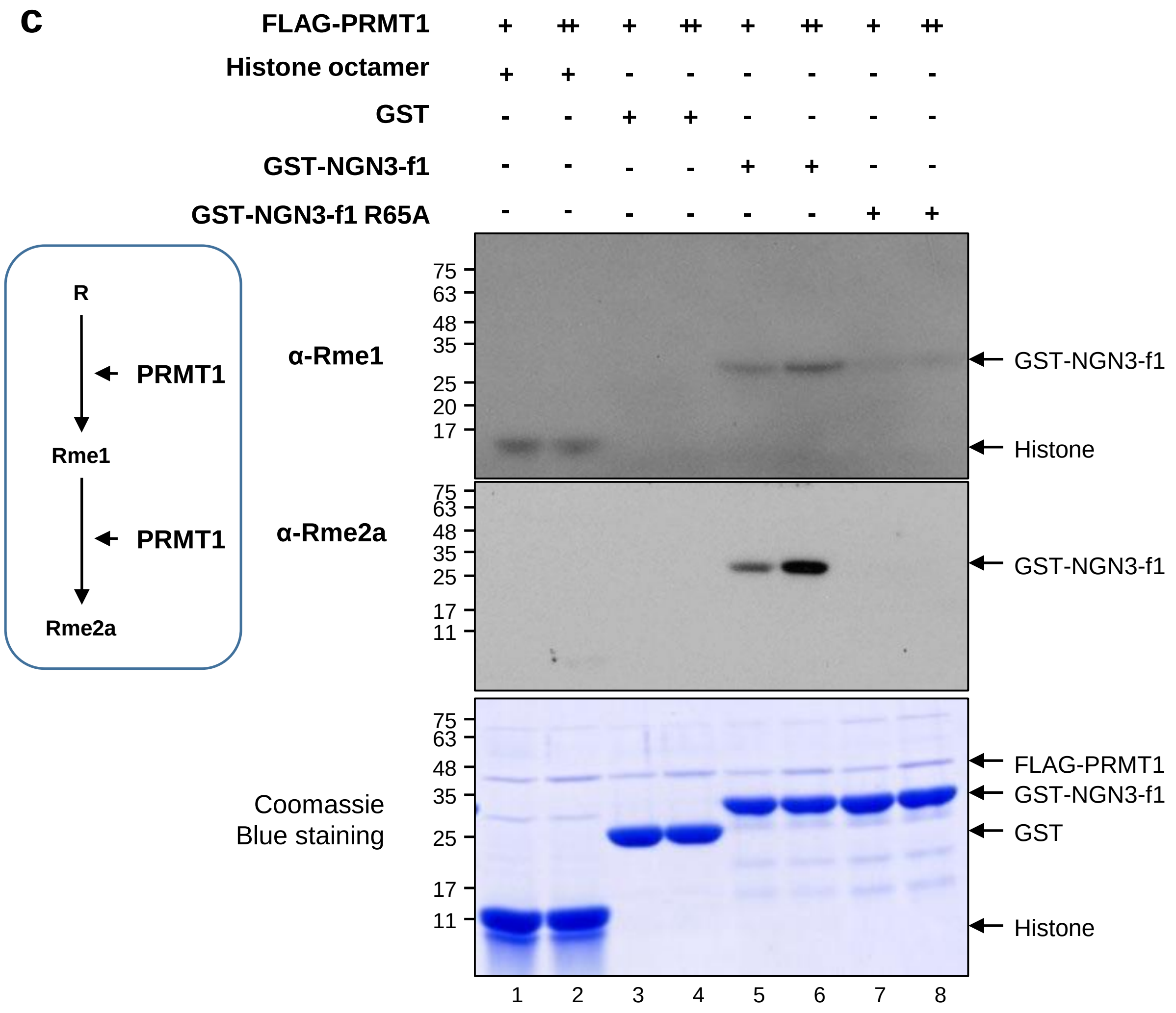


**Supplementary Fig. 6 Arginine methylation of NGN3 by PRMT1, *related to Fig. 4***

(a) Coomassie staining of GST-tagged NGN3 f1, f2 (asterisk), f1 R65A mutant (left), and mouse PRMT family members (right). f, fragment of NGN3; P, PRMT.

(b) Methylation of GST-tagged NGN3 f1 and f1 R65A mutant by mouse PRMT family members. Only PRMT1 methylated an arginine residue in NGN3 f1 (arrow).

(c) Methylation of GST-NGN3 f1 and R65A mutant by PRMT1. α-Rme1, mono-methylated arginine; α-Rme2a, asymmetrically di-methylated arginine. FLAG-PRMT1: +, 100 ng; ++, 200 ng. GST-NGN3 f1 and R65A: +, 400 ng, respectively.

# Supplementary Fig. 7

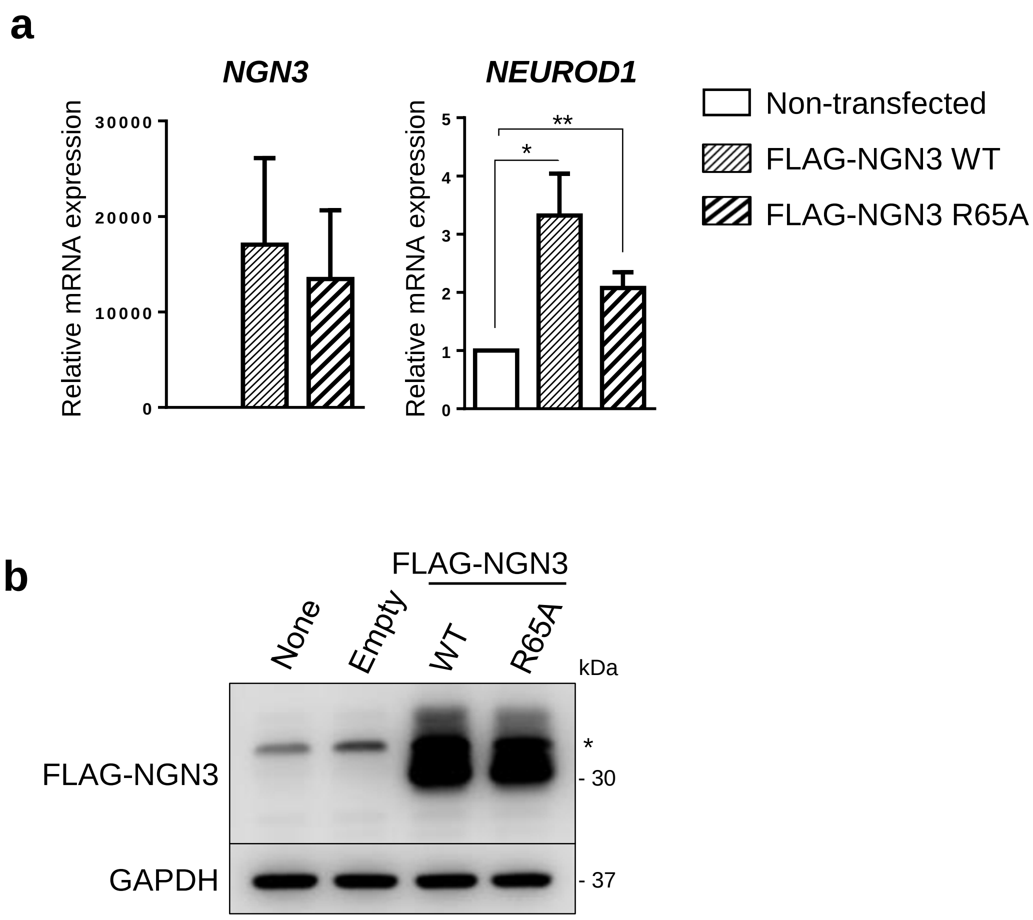


**Supplementary Fig. 7 Expression of NGN3 and *NEUROD1* in HEK cells**

(a) Relative mRNA expression of *NGN3* and *NEUROD1* in HEK cells transfected with pCAG-FLAG-NGN3 WT and R65A mutant. *$p$ < 0.05, **$p$ < 0.01. Data are represented as mean ± SEM (n = 4).

(b) Western blot analysis of pCAG-FLAG-NGN3-transfected HEK cells. “None” indicates cells incubated without any DNA vectors; “Empty” indicates cells transfected with pCAG-FLAG empty vector. The asterisk (*) indicates nonspecific bands.

# Supplementary Fig. 8

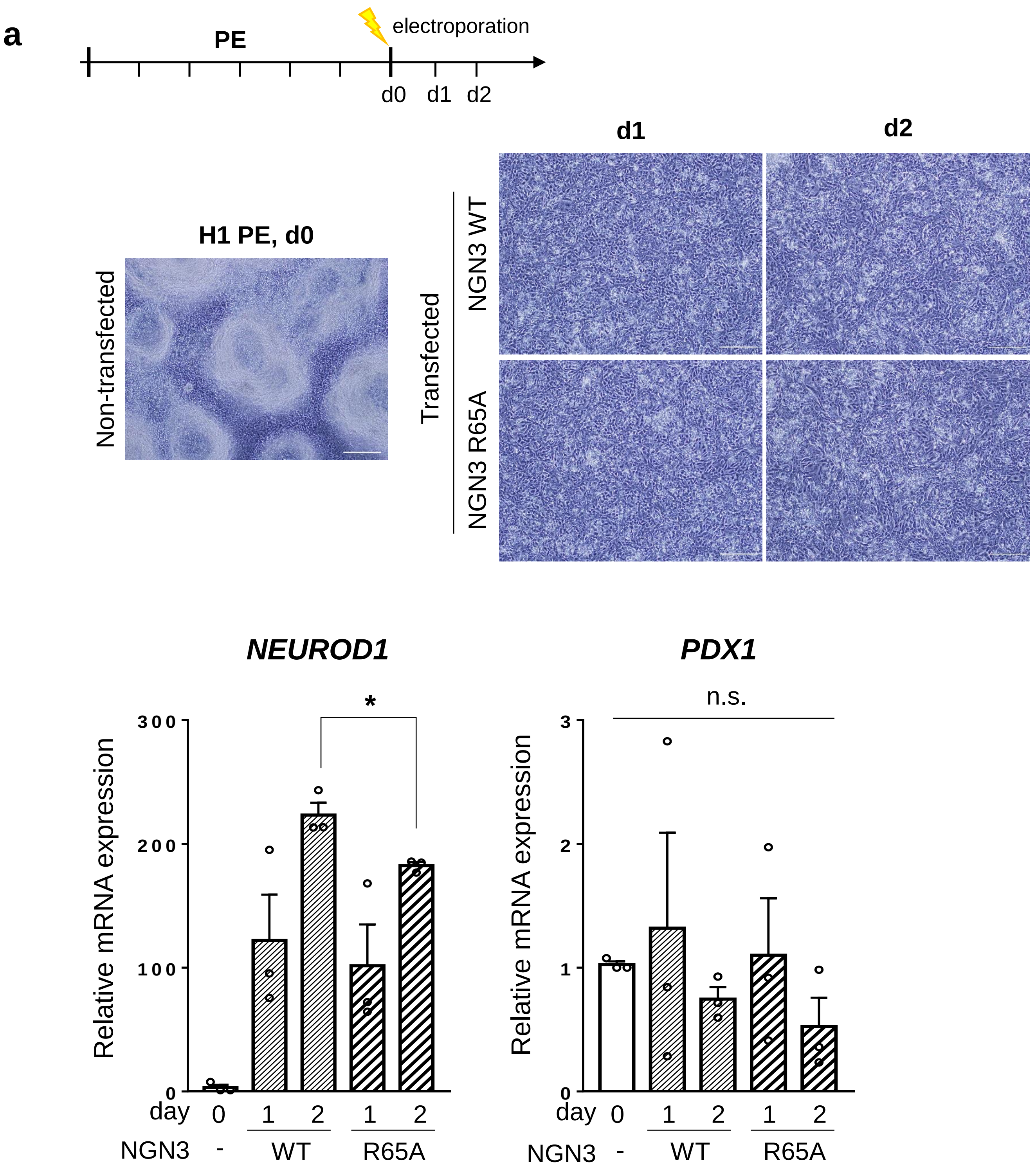

# Supplementary Fig. 8

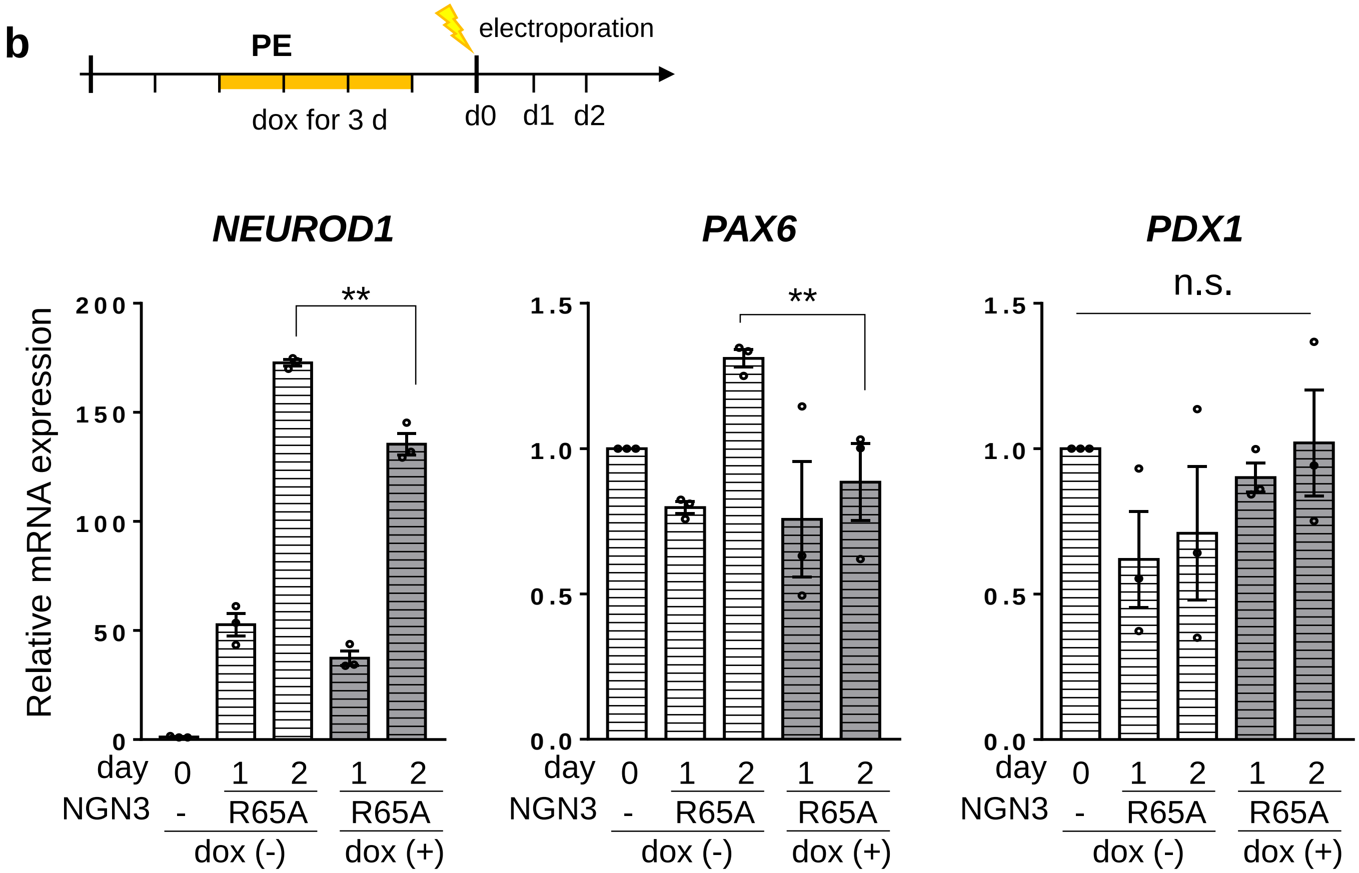


**Supplementary Fig. 8 Expression of *NEUROD1* in hESC-derived PE cells transfected with NGN3 R65A mutant**

(a) Bight field images of H1 PEs (transfected with NGN3 WT and R65A vectors, Scale bars, 200 μm) and relative mRNA expression of NEUROD1 and PDX1 genes in NGN3 transfected H1 PEs. n.s., not significant. *$p < 0.05$. Data are represented as mean ± SEM (n = 3).
(b) Relative mRNA expression of NEUROD1, PAX6 and PDX1 genes in dox (-) and dox (+) P-iKO PEs transfected with NGN3 R65A mutant. n.s., not significant. **$p < 0.01$. Data are represented as mean ± SEM (n = 3).

# Supplementary Fig. 9

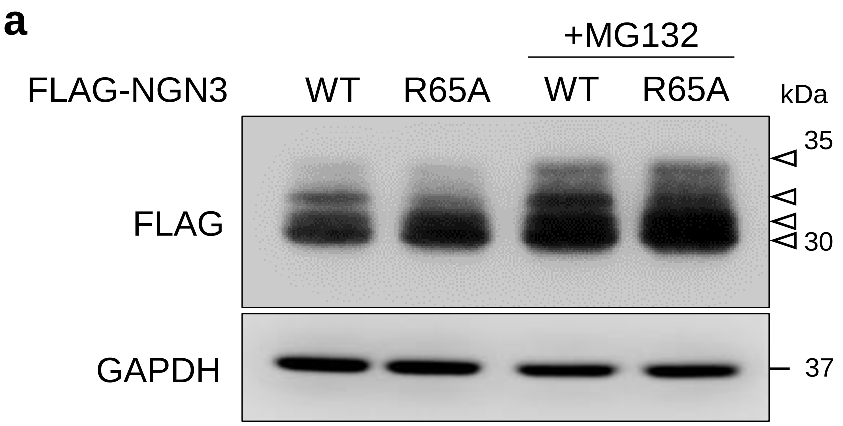


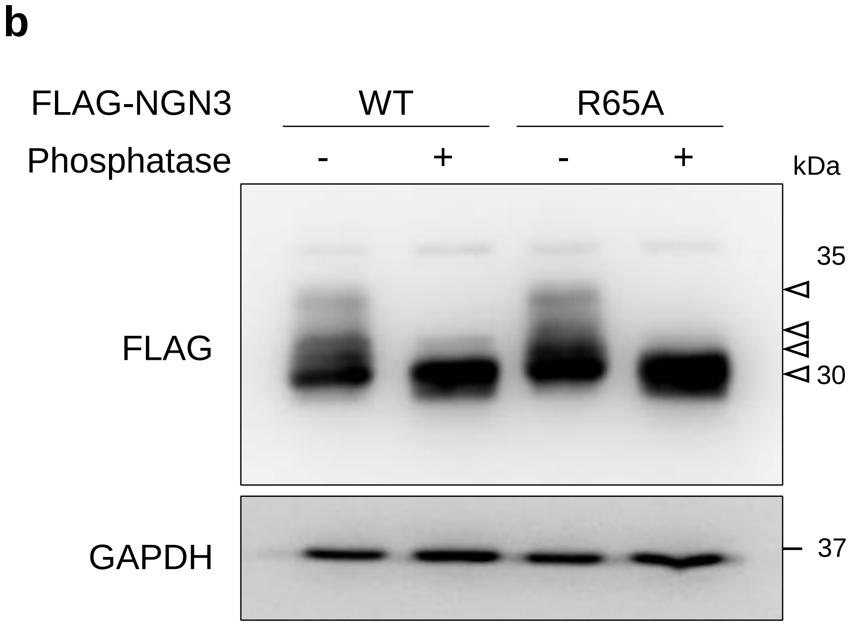


**Supplementary Fig. 8 Phosphorylation of NGN3 WT and R65A mutant, *related to Fig. 5***

(a) Western blot analysis of HEK cells transfected with FLAG-NGN3 WT and R65A mutant. Multiple NGN3 bands (arrowheads) were detected for both the WT and R65A mutant.

(b) Reduced NGN3 phosphorylation bands for both WT and R65A mutant induced by phosphatase treatment. Phosphorylated NGN3 bands (arrowheads) disappeared after phosphatase treatment (+).

## Supplementary Methods

### NGN3-T2A-EGFP donor plasmid

For NGN3-EGFP reporter cell line, NGN3 homology arm (HA)-EGFP vector and sgRNA expressing pRG2 vector were constructed. The sgRNA sequence for genomic NGN3 cleavage was 5'-GGGTCGCTCCTCCAGCGACG-3' [2]. The NGN3 5' HA and 3' HA was cloned from genomic DNA of H1 ESCs using nPfu-Forte (Enzynomics). Flag-T2A, eGFP sequences were taken from TLCV2 (#87360, Addgene, Watertown, MA, USA). The 5' HA of NGN3 has silent mutation to delete PAM sequence of NGN3 sgRNA. The stop codon of NGN3 was also eliminated.

### Generation of NGN3-T2A-EGFP hESCs

P-iKO ESCs were electroporated with 5ug of pRG2-sgRNA vector and 5ug NGN3 HA-EGFP vector. After electroporation, the quarter of cuvette were placed on Matrigel-coated 60mm dish. The dispersed cells were colonized in a week and each colony was transferred to multi-well plate for genotyping and further maintenance. HDR efficiency was 11.9% in hetero, and 2.38% in homo knock-in. In all experiment, homo NGN3-EGFP knock-in cell line was used.

## Supplementary References


1. Snijders, K.E., Cooper, J.D., Vallier, L. & Bertero, A, Conditional gene knockout in human cells with inducible CRISPR/Cas9. *Cris. Gene Ed*. 1–24. (2019)
2. Krentz, N. A. J. et al. Phosphorylation of NEUROG3 Links Endocrine Differentiation to the Cell Cycle in Pancreatic Progenitors Graphical Abstract HHS Public Access. *Dev. Cell* **41**, 129–142 (2017)
